# THY-1 Cell Surface Antigen (CD90) Has an Important Role in the Initial Stage of Human Cytomegalovirus Infection

**DOI:** 10.1371/journal.ppat.1004999

**Published:** 2015-07-06

**Authors:** Qingxue Li, Adrian R. Wilkie, Melodie Weller, Xueqiao Liu, Jeffrey I. Cohen

**Affiliations:** 1 Medical Virology Section, Laboratory of Infectious Diseases, National Institutes of Health, Bethesda, Maryland, United States of America; 2 Secretory Physiology Section, Molecular Physiology and Therapeutics Branch, National Institutes of Health, Bethesda, Maryland, United States of America; University of Alabama at Birmingham, UNITED STATES

## Abstract

Human cytomegalovirus (HCMV) infects about 50% of the US population, is the leading infectious cause of birth defects, and is considered the most important infectious agent in transplant recipients. The virus infects many cell types in vivo and in vitro. While previous studies have identified several cellular proteins that may function at early steps of infection in a cell type dependent manner, the mechanism of virus entry is still poorly understood. Using a computational biology approach, correlating gene expression with virus infectivity in 54 cell lines, we identified THY-1 as a putative host determinant for HCMV infection in these cells. With a series of loss-of-function, gain-of-function and protein-protein interaction analyses, we found that THY-1 mediates HCMV infection at the entry step and is important for infection that occurs at a low m.o.i. THY-1 antibody that bound to the cell surface blocked HCMV during the initial 60 minutes of infection in a dose-dependent manner. Down-regulation of THY-1 with siRNA impaired infectivity occurred during the initial 60 minutes of inoculation. Both THY-1 antibody and siRNA inhibited HCMV-induced activation of the PI3-K/Akt pathway required for entry. Soluble THY-1 protein blocked HCMV infection during, but not after, virus internalization. Expression of exogenous THY-1 enhanced entry in cells expressing low levels of the protein. THY-1 interacted with HCMV gB and gH and may form a complex important for entry. However, since gB and gH have previously been shown to interact, it is uncertain if THY-1 directly binds to both of these proteins. Prior observations that THY-1 (a) interacts with αVβ3 integrin and recruits paxillin (implicated in HCMV entry), (b) regulates leukocyte extravasation (critical for HCMV viremia), and (c) is expressed on many cells targeted for HCMV infection including epithelial and endothelial cells, fibroblast, and CD34+/CD38- stem cells, all support a role for THY-1 as an HCMV entry mediator in a cell type dependent manner. THY-1 may function through a complex setting, that would include viral gB and gH, and other cellular factors, thus links virus entry with signaling in host cells that ultimately leads to virus infection.

## Author Summary

Human cytomegalovirus (HCMV) is an important human pathogen that infects about half the US population and is a major cause of birth defects and morbidity in transplant recipients. Despite extensive research, much is still unknown regarding how the virus enters cells. We identified THY-1, a protein on the surface of many different cell types susceptible to CMV infection, as having an important role for facilitating virus infection. We found that antibody to THY-1 or soluble THY-1 protein blocked HCMV infection in multiple cell types, suggesting that THY-1 might serve as a potential therapeutic target to reduce infection. Expression of exogenous THY-1 increased susceptibility of cells to HCMV infection. We showed that THY-1 has an important role in a host signaling pathway that is initiated when HCMV infects cells. Furthermore, we found that THY-1 interacted with HCMV glycoproteins that initiate entry of virus into the cell. THY-1 is known to interact with several host cell proteins important for infection and is expressed on numerous types of cells that can be infected by HCMV. Thus, we have identified THY-1 as a molecule that has an important role in the initial stage of HCMV infection.

## Introduction

Human cytomegalovirus (HCMV) infects about 50% of the US population and is the leading infectious cause of birth defects and the most important infectious agent in transplant recipients. In vivo, HCMV predominantly infects epithelial, endothelial, fibroblast, smooth muscle, and mononuclear cells including myeloid progenitors and dendritic cells [1,2]. Primary infection typically begins with virus replication in mucosal epithelium followed by leukocyte-associated viremia. Among more than 50 putative glycoproteins encoded by HCMV, gH/gL and gB are conserved in the herpesvirus family, and are required for HCMV entry into cells [3]. gH and gL interact with UL128-UL131 proteins to form a pentameric complex or with gO to form a trimer, that are important for infection of different cell types [4–6]. gB has been reported to bind to gH/gL, and functions as a fusogen [7,8]. In addition, gB binds to heparan sulfate proteoglycans [9–11].

HCMV initiates infection by attachment to cell surface heparan sulfate proteoglycans [12,13] followed by engagement of cellular receptors or entry mediators. Previous studies have identified several cellular proteins that may function at early steps of infection, including platelet-derived growth factor receptor-α (PDGFR-α) [14,15], epidermal growth factor receptor (EGFR) [16], DC-SIGN [17], αVβ3 and β1 integrins [18,19], and paxillin [20]. HCMV, like many other viruses, utilizes host molecules to facilitate entry in a cell type dependent manner. gB and gH interact with these cellular molecules [14,16–18,21,22]; however, it is not clear whether the interactions are direct or indirect through protein complexes that may include various viral and cellular components. Virus entry is not only limited to virion internalization and cell signaling is an integral part of the entry process [23]. Previous work has shown that HCMV induced activation of the Akt signaling pathway is required at an early step in virus entry [22]. HCMV utilizes PDGFR- α to facilitate entry and simultaneously induces phosphorylation of PDGFR- α when the virus infects fibroblasts, endothelial and epithelial cells. The virus activates EGFR when it infects monocytes, and employs integrins and paxillin at the beginning of infection. The activation of either PDGFR- α or EGFR in turn leads to activation of downstream cellular phosphatidylinositol 3-kinase (PI3K), Src kinase and focal adhesion kinase (FAK) signaling pathways, and induces cytoskeletal rearrangements to create an intracellular environment to facilitate infection [14,20].

HCMV infects a broad spectrum of human cells ranging from epithelial and endothelial cells to hepatocytes and neuronal cells. This may reflect the capability of the virus to utilize multiple cellular molecules to gain entry depending on the type of cell. The observation that cells expressing neither PDGFR- α nor EGFR are still permissive for HCMV infection implies that the virus exploits additional host factors at an early step of infection [3,24]. In an attempt to identify other cellular proteins important for infection, we utilized 54 human cell lines from the NCI-60 panel of diverse tissue origins whose gene expression profiles have been extensively analyzed across multiple platforms [25–27]. A previous study showed that transcript-protein correlation in these cell lines is highly statistically significant [28]. In conjunction of bioinformatics analysis, this panel of cell lines has been a valuable screening tool for identifying host factors important for viral infection [29–33]. We investigated the susceptibility of these cell lines for HCMV and correlated infectivity with gene expression profiles for each of the cell lines using bioinformatics analysis. This approach allowed us to evaluate the contribution of individual host molecules to infection in the context of overall gene expression in the cells. We focused on membrane associated proteins since they are likely to be involved in the very early steps of virus infection. Using a series of loss-of-function, gain-of-function and ligand interaction analysis, and additional non-transformed cells, the biological function of one candidate protein was further validated. Here, we report that THY-1 has an important role in the early stages of HCMV infection in a diverse group of cell lines.

## Results

### Identification of THY-1 as a putative host determinant for HCMV infection using a computational biology approach

Prior studies to identify entry mediators for HCMV have been limited by the types of viruses and cell lines used. High passage strains of HCMV, deleted for the UL128-131 region, which are restricted to efficient growth in fibroblasts, have been predominately used to identify HCMV entry mediators [14,16,22]. In addition, previous studies defining HCMV entry used relatively few cell lines, and most studies focused on fibroblasts. Since HCMV utilizes different host molecules to infect specific types of target cells (mainly endothelial, epithelial, and mononuclear cells in vivo), these more traditional approaches with one or only a few cell lines have limitations. To address the issue, we utilized a panel of 54 adherent cell lines of diverse origins from the NCI-60 panel whose molecular profiles have been extensively characterized at the DNA, RNA and protein levels, and integrated with each other by integromic analyses [26,34] (S1 Table). We infected the cells with both fibroblast (Towne-GFP) (a gift from Dr. H. Zhu, UMDNJ-New Jersey Medical School) and epithelial/endothelial tropic (BADrUl131-GFP and TB40E-GFP) HCMV which express GFP (gift from Dr. T. Shenk, Princeton University) [4,35–37]. Two or three days post infection, susceptibility to HCMV was determined based on GFP positivity of the cells. For bioinformatics analyses, infection of each cell line with each virus was performed in at least three independent experiments and each time in triplicate wells. Infectivity was then determined by FACS analysis of GFP positive cells and the mean infectivity score was calculated by normalization using epithelial (ARPE-19) cells for epithelial/endothelial tropic virus and fibroblasts (MRC-5 cells) for fibroblast tropic virus (S2 Table). Correlations between HCMV infectivity and expression of each cellular gene were calculated using the COMPARE algorithm [38] and further detailed using MAPP software [30]. COMPARE utilizes gene expression profiling as determined by microarray analysis across multiple microarray platforms to identify genes that correlate (based on the Pearson Correlation Coefficient) with the experimentally determined HCMV infection profile [30,38]. The mean infectivity score and the expression level of each gene were computed and the Pearson Correlation Coefficient was determined.

**Fig 1:**
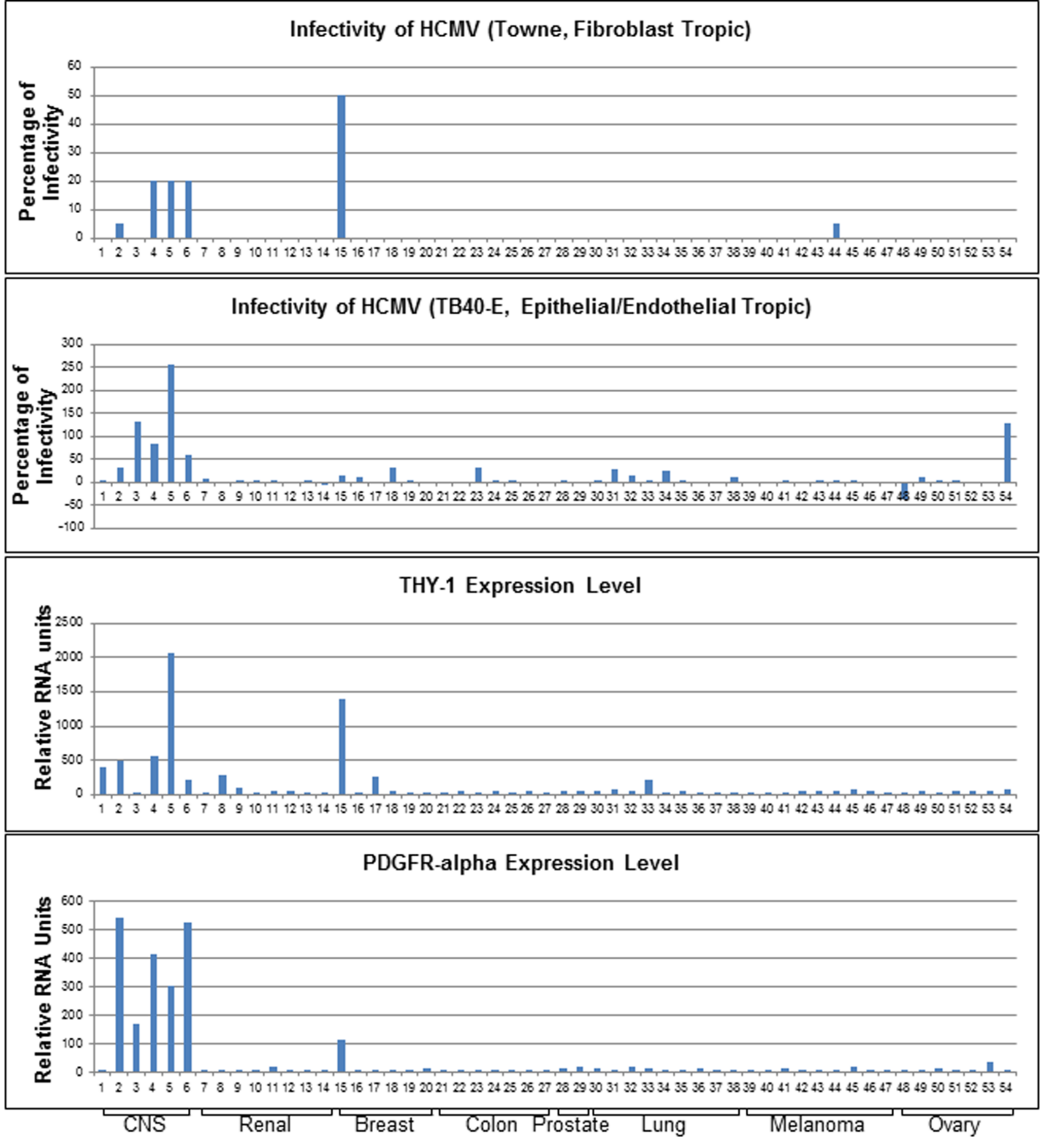
Expression of THY-1 positively correlates with HCMV infectivity for both fibroblast and epithelial/endothelial tropic stains of virus in 54 human cell lines. Cells were infected with both fibroblast tropic HCMV (Towne-GFP) for 2 days and epithelial/endothelial tropic HCMV (TB40E-GFP) for 3 days. We aimed to achieve an infection rate at approximately 10–20% for positive controls without acid inactivation (m.o.i 0.5 for control MRC-5 cells and 0.1–0.5 for ARPE-19 cells). The percentage of infectivity was derived from the mean of three independent infections minus the background florescence contributed by mock-infected cells and normalized against control cell lines (MRC-5 for Towne-GFP, ARPE-19 for TB40E-GFP). THY-1 and PDGFR-α expression levels were determined by multiple microarray platforms (http://dtp.nci.nih.gov/mtweb) and these are baseline levels of RNA in cells not infected with virus. HCMV infectivity positively correlated with THY-1 expression at a level similar to that of PDGFR-α. Cell lines are indicated in S1 Table.

The highest rated membrane associated protein whose expression correlated positively with virus susceptibility was PDGFR-α, which has been shown to function in HCMV entry [14,15]. Transfection of MRC-5 cells with PDGFR-α specific siRNAs reduced HCMV infection (S1A Fig). THY-1 was implicated as the next highest scoring membrane associated protein whose expression correlated positively with HCMV infectivity. Infectivity of both epithelial/endothelial and fibroblast tropic HCMV strains showed a positive correlation with THY-1 expression at a level similar to or higher than that of PDGFR-α (Fig 1). The correlation of THY-1 expression was statistically significant for both Towne-GFP (P = 0.0002, Pearson Correlation Coefficient 0.46) and TB40E-GFP HCMV (P = 0.0004, Pearson Correlation Coefficient 0.44). Likewise, expression of PDGFR-α correlated with infection for Towne-GFP (p<0.00001, Pearson Correlation Coefficient 0.53) and TB40E-GFP HCMV (p = 0.016, Pearson Correlation Coefficient 0.29). Similar correlations for THY-1 and PDGFR-α expression with infectivity were also observed for epithelial/endothelial tropic strain BADrUl131-GFP HCMV.

### Soluble THY-1 protein blocks HCMV infection during, but not after, virus internalization

**Fig 2:**
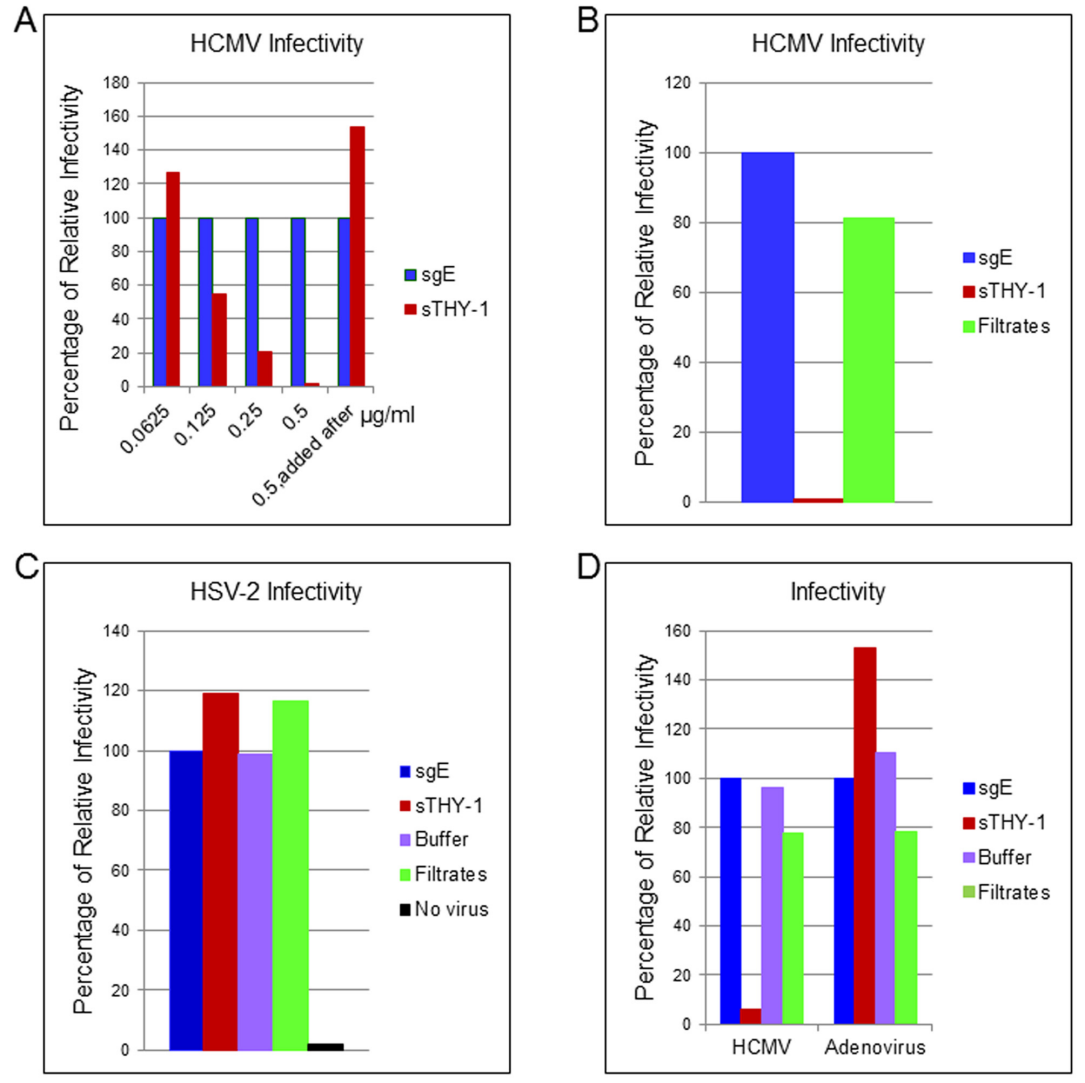
Soluble THY-1 protein blocks HCMV entry in a dose-dependent manner. (A) HS-578T (adenocarcinoma) cells were infected with Towne-GFP in the presence of increasing amounts of soluble protein. The virus was allowed to enter for 60 min as described in the legend to Fig 2. After culture for 3 days GFP-positivity was quantified by FACS. Equal amounts of THY-1-His and gE-His proteins were used in the assays based on Micro BCA Protein analysis (Pierce, Rockford, IL) and ELISA to determine “His” units [46]. Seven independent experiments were performed with P value < 0.0001. (B) Towne-GFP HCMV was added to MRC-5 cells at an m.o.i. of 0.1 in the presence of soluble THY-1 (0.25 ug/ml) or control protein sgE (soluble varicella-zoster virus derived gEt-His) or control filtrates obtained during purification of THY-1 protein with an Amicon Ultra centrifugal filter unit (3000 molecular weight cutoff). The virus was allowed to enter the cells for 60 min at 37°C as indicated above followed by low pH citrate buffer wash to inactivate non-internalized virus and remove the soluble proteins. Infectivity was determined by FACS analysis of GFP positive cells at day 3 post-infection. (C) HS-578T cells were infected with HSV-2-GFP (m.o.i. 0.5) in the presence of soluble THY-1 protein (0.5 μg/ml), a control protein (soluble varicella-zoster virus gE, 0.5 μg/ml) or filtrates (derived from THY-1 protein purification in which THY-1 protein was removed by an Amicon Ultra filtration column with a 3000 molecular weight cutoff). Virus infection was performed as described in (A) and then overlaid with 2% human intravenous immune globulin (IVIG) (Talecris Biotherapeutics, Research Triangle Park, NC) and GFP positive cells were determined at 24 hrs post-infection by FACS. (D) HS-578T cells were infected with Towne-GFP or Adenovirus-GFP (0.5–1.0 m.o.i.) in the presence of soluble proteins (0.5 μg/ml), filtrate control, or buffer for 60 min as described in the legend to Fig 2, and the percentage of infected cells was determined by flow cytometry 3 days after infection.

To determine whether THY-1 is important for HCMV infection, we performed a series of loss-of-function experiments. First, we determined if soluble THY-1 (a.a. 20–130) can block HCMV infection. Wild-type THY-1 is initially synthesized as a 161 amino acid peptide. Upon maturation, the signal peptide (a.a.1-19) is cleaved and the C-terminal a.a. 132–162 is replaced with a GPI anchor. A soluble form of THY-1 (a.a. 20–130) exists in vivo and the recombinant form of THY-1 retains its biological function in binding integrins [39,40]. HCMV or control virus (HSV-2-GFP or adenovirus-GFP) was premixed with soluble THY-1-His protein or a control His protein (soluble varicella-zoster virus gE-His) at room temperature for 10 min, added to HS-578T cells for virus binding on ice for 60 min. Internalization was initiated by raising the temperature to 37°C for 60 min, and then non-absorbed virus was inactivated at low pH, and infectivity was quantified using GFP 3 days later. Compared with the control protein at each dose, soluble THY-1 protein reduced HCMV infectivity in a dose-dependent manner (Figs 2A and S3) in adenocarcinoma cells, and inhibited infection in MRC-5 fibroblasts (Figs 2B and S4 top). In contrast, it did not reduce HSV-2 infectivity (Figs 2C and S4 bottom) or adenovirus infectivity (Figs 2D and S4 Bottom). Soluble THY-1 protein was required during the initial viral entry step to block HCMV infectivity, since addition of the protein after virus binding and internalization did not inhibit infectivity (Fig 2A, last bar). In natural hosts HCMV infection likely occurs at a relatively low m.o.i. A review of studies of virus shedding from saliva of infants, children, and adults, often the source of transmitted virus, showed that the titer of virus in saliva ranged from 10^3^ to 2 x 10^4^ pfu/ml [41]. Therefore we infected the cells with titers ranging from 4 x 10^4^ (HS-578T) to 1 x 10^5^ pfu/ml (MRC-5), which corresponds to a relatively low m.o.i. (0.05 to 1) to try to replicate what may occur during natural infection. Furthermore, we used acid inactivation to limit the infection within the first 60 min to focus on the initial stages of virus infection and the most efficient pathways for viral entry (Fig 2). During the first 60 minutes after infection (m.o.i. 0.05–1 with acid inactivation), about 2–10% of the cells were infected, which corresponds to about 20–35% of the cells if the same infection is allowed to continue for a prolonged time, i.e. without acid inactivation (S5 Fig). Soluble THY-1 protein blocked over 90% of the infection that occurred within the first 60 min (m.o.i. 0.05–1) at a dose of 0.5 μg/ml (Fig 2A). In contrast, with a high m.o.i (4, based on titration in MRC-5 cells) 10-fold more soluble protein was required to block >90% of the infectivity (during entry over 60 min with acid inactivation), and soluble THY-1 blocked infection less efficiently for the virus that enters with slower kinetics (75% reduction in infectivity without acid inactivation, S6 Fig).

### THY-1 antibody blocks HCMV infection during the initial 60 minutes of infection in a dose-dependent manner

**Fig 3:**
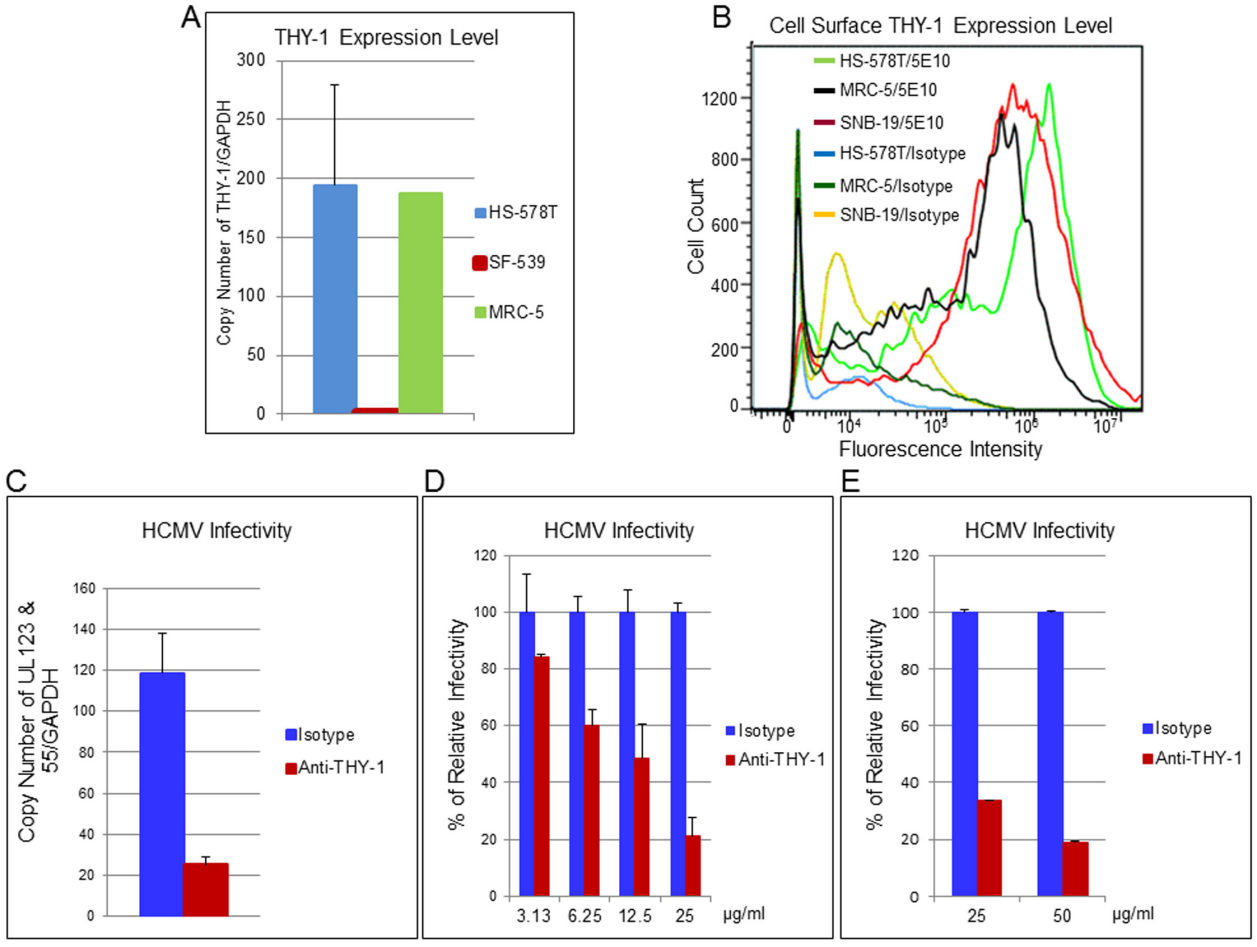
THY-1 antibody binds to cell surface THY-1 protein and blocks HCMV entry in a dose-dependent manner. (A) Total RNA was extracted from different cell lines as described in the Materials and Methods. Quantitative real-time RT-qPCR was performed targeting THY-1 (FAM labeling) and normalized against GAPDH (VIC labeling) which was amplified in the same wells. (B) Cells were incubated with THY-1 specific monoclonal antibody 5E10 or isotype control on ice for 60 min. After washing with cold PBS, the cells were stained with anti-mouse-Alexa-488 conjugate on ice for 30 min, washed, fixed with 2% paraformaldehyde, and analyzed by FACS. (C)–(E) Anti-THY-1 antibody (5E10) or isotype control (25 μg/ml) was added to cells for 60 min on ice for surface binding and then the antibody was washed off. HCMV was added at 0.05 m.o.i. for 60 min at 4°C to allow virus binding, and the temperature was shifted to 37°C for 60 min to allow virus entry. The remaining virus that had not internalized was then inactivated with low pH citrate buffer, and the cells were washed twice with cell culture medium. (C) HS-578T (adenocarcinoma) cells were infected with Towne-GFP virus. RNA was extracted at 6 hr post-infection and HCMV UL123 and UL55 mRNAs were quantified using RT-PCR and normalized against GAPDH mRNA amplified in the same reaction. The experiment was performed 4 times in triplicate, and a representative result is shown here. (D) HS-578T cells were infected with HCMV as described above after incubation with different concentrations of antibody with the cells. After culture for 3 days, GFP-positivity was determined by FACS. The experiment was performed three times in triplicate. (E) MRC-5 cells were pre-incubated with THY-1 antibody 5E10 or IgG control at 25 or 50 ug/ml as described above and infected with TB40E-GFP virus for 60 min before low pH citrate buffer inactivation. Infectivity was assayed for expression of GFP by FACS at 3 days post-infection.

Next, we examined whether specific antibody 5E10 binds to cell surface THY-1 protein. NCI-60 cell lines SNB-19 (glioblastoma) and HS-578T (adenocarcinoma), as well as primary human diploid (MRC-5) fibroblasts all express THY-1 mRNA [34] (Fig 3A), and THY-1 protein was detected on the surface of these cells (Figs 3B and S1B). Both HS-578T and SNB-19 cells support productive HCMV infection and produce progeny virus (S2 Fig), although HCMV cell-to-cell spread in SNB-19 cells is limited, especially with TB40E-GFP HCMV. To ascertain whether THY-1 specific antibody blocks HCMV infection, THY-1 or isotype control antibody was allowed to bind to the surface of HS-578T cells on ice for 60 min, the antibody mixture was removed from the cells, and HCMV was added on ice for 60 min to synchronize virus binding. To focus on the early steps of virus entry, the temperature was raised to 37°C for 60 min to allow virus entry, followed by low pH treatment to inactivate any virions that still remained on the cell surface or in the medium. After washing, the cells were then cultured for 6 hr before RNA extraction to quantify combined HCMV UL123 (encodes IE1) and UL55 (encodes gB) RNA expression by RT-qPCR [42] or for 3 days to measure infectivity by FACS for GFP. Although UL55 is a late gene, UL55 transcripts start to appear at 4 hrs post-infection, and expression is not strictly dependent on new viral DNA synthesis [43,44]. In 4 independent experiments, quantitative RT-PCR showed that THY-1 specific antibody blocked expression of HCMV UL123 and UL55 genes, compared with isotype control antibody (Fig 3C, P = 0.0002 for 4 independent experiments). Similar blocking result with THY-1 antibody was also seen when infectivity was assayed at 3 days post-infection by virus-encoded GFP (Fig 3D, P = 0.0004, 3 independent experiments). THY-1 specific antibody, but not isotype control, blocked HCMV infectivity in a dose-dependent manner (Figs 3D and S7). THY-1 antibody also blocked HCMV infection in primary MRC-5 cells (Fig 3E).

### Down-regulation of THY-1 expression impairs HCMV infectivity during virus entry

**Fig 4:**
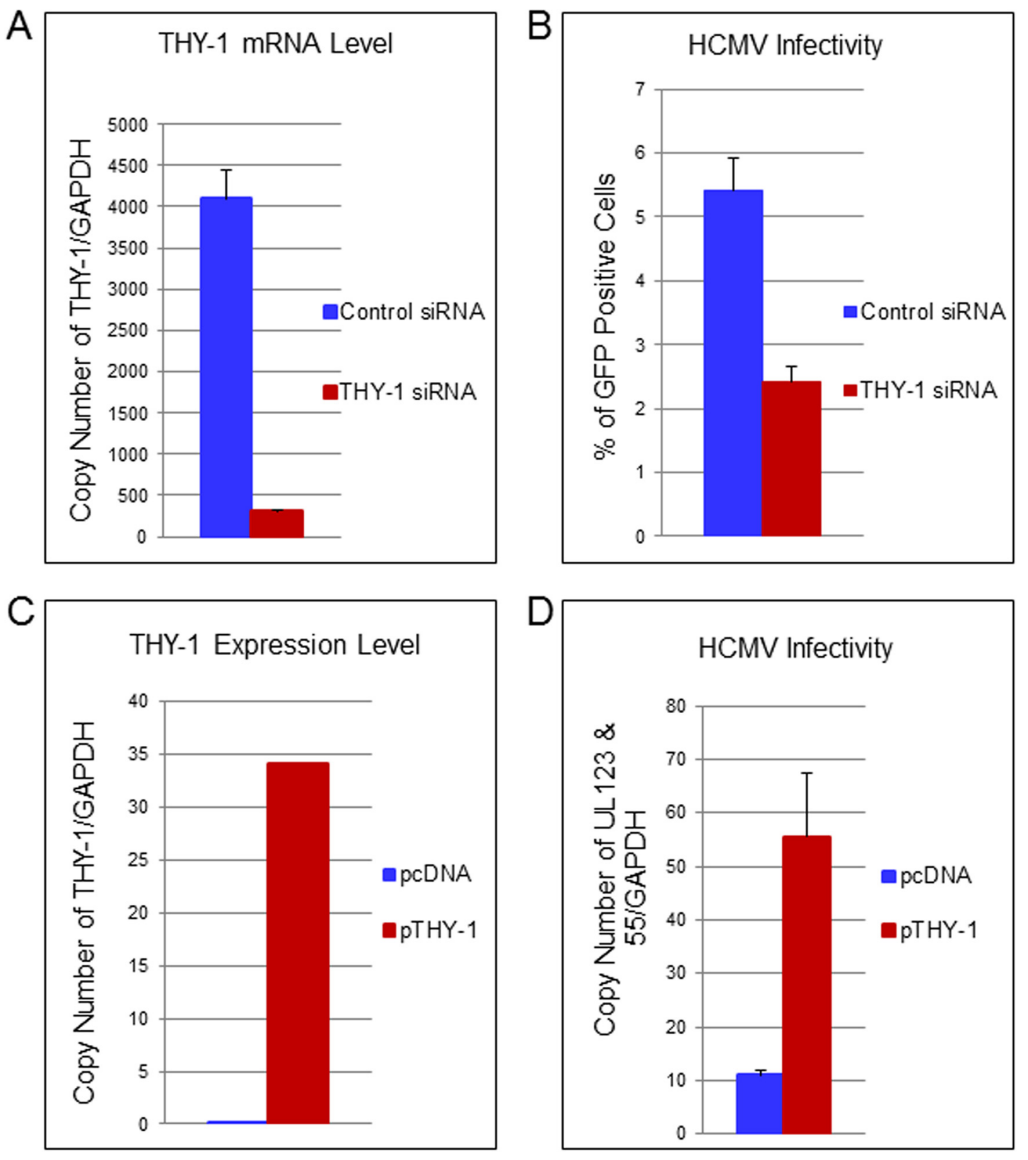
Down-regulation of THY-1 expression impairs HCMV entry and exogenous expression of THY-1 enhances entry. (A) Quantitative RT-PCR of THY-1 expression in SNB-19 (glioblastoma) cells 48 hr after nucleofection of THY-1 specific siRNAs and control non-targeting siRNAs. THY-1 specific siRNAs knocked down THY-1 expression. (B) HCMV infectivity of SNB-19 cells treated with siRNAs for 48 hr and infected with epithelial/endothelial tropic HCMV TB40E-GFP (m.o.i. 0.1). The virus was incubated with cells for 60 min to allow entry before inactivation of virus that had not internalized, and GFP-positive cells were quantified by FACS at day 3 post-infection. Down-regulation of THY-1 by specific siRNAs inhibited HCMV infectivity (P <0.001). 14 independent experiments were performed and a representative result is shown. (C) SF-539 cells were nucleofected with a plasmid expressing THY-1 (pCMV-THY1) or vector control. At 48 hrs post-transfection THY-1 mRNA was quantified by RT-PCR and normalized to GAPDH mRNA amplified in the same reaction. (D) SF-539 cells were infected with Towne-GFP at 48 hr post-transfection for 6 hr and HCMV UL123 and UL55 mRNAs were quantified by RT-PCR and normalized against GAPDH mRNA. Seven independent experiments were carried out and a representative experiment is shown.

To confirm the loss-of-function findings observed with THY-1 specific antibody, we used THY-1 specific siRNAs to knockdown THY-1 expression in permissive cells, and analyzed the effect on HCMV infection. Nucleofection of cells with THY-1 specific siRNAs reduced THY-1 expression by over 90% compared with control siRNAs at the time of infection both at the mRNA (Fig 4A) and protein level (S10A Fig and see section on THY-1 and Akt activation below). HCMV infectivity was reduced by 30–50% at 3 days post-infection based on FACS analysis for GFP (Fig 4B) (P value <0.0001, 12 independent experiments). However, THY-1 siRNAs knocked down cell surface THY-1 protein (S10B Fig) less effectively than total THY-1 protein (S10A Fig). This might be due to increased stability of surface THY-1 protein when it is anchored into lipid rafts, and could contribute to the lower level of inhibition of HCMV infection with THY-1 siRNAs than with antibody or soluble protein (see above). The impairment of HCMV infectivity following knockdown of THY-1 was observed in glioblastoma (SNB-19), adenocarcinoma (HS-578T) and MRC-5 cells infected with either epithelial/endothelial or fibroblast tropic HCMV. Since the infection protocol allowed only 60 min for virus entry before virus was inactivated by low pH, the reduction of infectivity occurred during initiation of HCMV infection.

### Expression of exogenous THY-1 enhances HCMV entry into cells

In contrast with SNB-19 and HS-578T cells which support HCMV infection and express THY-1 on their surface (used above in loss-of-function experiments), SF-539 (gliosarcoma) cells express negligible levels of THY-1 mRNA or THY-1 protein on the cell surface (Figs 3A and S1B), and are refractory to HCMV Towne infection. Molecular profiling of NCI-60 cells showed that SF-539 cells express comparable levels of PDGFR-α, EGFR, αVβ3 and β1 integrins as SNB-19 and HS-578T cells. Therefore, we used SF-539 cells for gain-of-function studies. pCMV-THY-1 or empty vector was transfected into SF-539 cells by nucleofection and 48 hr later the cells were incubated with Towne-GFP for 1 hr on ice, then at 37°C for 1 hr, followed by low pH to inactivate virus that had not entered the cells. RNA was extracted from the cells at the time of infection to monitor THY-1 expression and at 6 hrs post-infection to detect HCMV UL123 and UL55 expression. Quantitative RT-PCR showed that SF-539 cells transfected with control vector expressed very low levels of THY-1 mRNA, while cells transfected with pCMV-THY-1 expressed high levels of THY-1 mRNA (Fig 4C). Expression of THY-1 from the pCMV-THY-1 plasmid enhanced HCMV infectivity of the cells (Fig 4D, P <0.0001, 7 independent experiments). Since the infection was restricted to the initial 60 min of viral inoculation, we conclude that exogenous expression of THY-1 enhances the initial stage of HCMV infection of cells.

### Pull-down of complex using THY-1 antibody or purified THY-1 protein indicates that THY-1 interacts with gB and gH

gB and gH/gL are essential for HCMV infection [3]. HCMV gB has a furin cleavage site that results in covalently bound N-terminal and C terminal fragments of about 55 kD each. gB has been reported to bind to gH and may form glycoprotein complexes with other components, including gO or UL128-131 [9,10,45]. We postulated that since THY-1 is important for HCMV infection, it might interact with one or more of these glycoproteins, either directly or as part of a complex. We incubatedanti-THY-1 or isotype control antibody with HCMV-infected and uninfected cell lysates, and separated the immune complexes by gel electrophoresis. Several protein bands were found in lysates from HCMV Towne infected MRC-5 cells immunoprecipitated with antibody to THY-1, but not in lysates immunoprecipitated with isotype control antibody or in uninfected cells. Mass spectrometry of these unique bands identified gB and gH with a Mascot score of 1141 and 281 (a score of 45 represents the significance threshold for individual peptide matches P <0.05), with multiple peptide sequence coverage for both glycoproteins. In contrast, gM and gO were each identified by only a single peptide (S3 Table).

**Fig 5:**
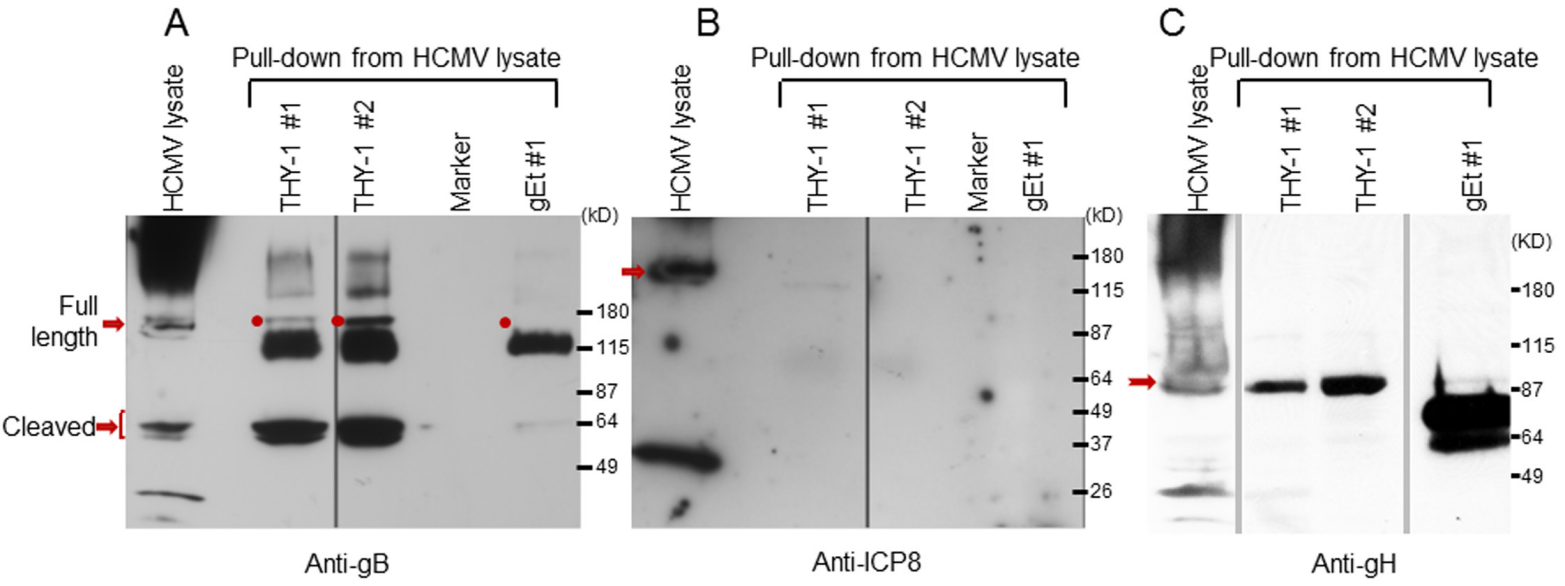
HCMV gB and gH, but not ICP8, obtained from infected cell lysates binds to a THY-1 protein column. (A) Anti-gB antibody detects full length gB (160 kD) and furin cleaved gB (55 kD doublets) in eluates of HCMV-infected cell lysates from THY-1 protein columns with either of two lysis buffers, but not from the VZV gE protein column. The very dark 115 kDa band seen in the THY-1 and gEt bands are background bands likely due to cross-reactivity of the anti-gB or secondary antibody with protein from the His column. (B) Anti-ICP8 antibody detects a 135 kD protein band in lysate from MRC-5 cells infected with HCMV AD169, but not from eluates of lysates applied to THY-1 or VZV gE protein columns. Infected cell lysates were prepared using lysis buffer 1 (PBS with 0.1% NP-40) or lysis buffer 2 (25 mM Tris, 15 mM NaCl and 0.1% NP-40). (C) Anti-gH antibody detects gH (92 kD) in eluates of HCMV-infected cell lysates from THY-1 protein columns with two different lysis buffers. gH was not detected in eluate from a control varicella-zoster gE column.

Since co-immunoprecipitation followed by Western blotting was inefficient for detecting proteins that interact with gB in infected cells, we constructed protein columns by binding THY-1-His protein, or control VZV gE-His protein to Talon beads, added lysates from HCMV-infected cells to the columns, eluted proteins bound to the columns, and immunoblotted the proteins with antibody to HCMV ICP8 or gB. Two different cell lysis buffers were used, PBS with 0.1% NP-40 [30] and 25 mM Tris, 15 mM NaCl and 0.1% NP-40 [46]. HCMV gB was detected in the infected cell lysate and in eluates from THY-1 protein columns, but not the control VZV gE protein column (Fig 5A). Interestingly, THY-1 complexed with full length gB (160 kD), as well as its proteolytic cleavage products of 55 kD [47]. Purified THY-1 protein pulled down more 55 kD gB than full length gB. A previous study has shown the cleaved form of gB was more abundant than full length gB in infected cell lysate and in purified virions [48]. In contrast, the 135 kD HCMV ICP8 was detected in infected cell lysate, but not in eluates from THY-1 or control VZV gE protein columns (Fig 5B). Similarly, gH was co-precipitated from infected cell lysate by purified THY-1 protein (Fig 5C). These results suggest that THY-1 may form a complex with HCMV gB and gH in infected cells. Since gB and gH have been shown to form a complex, it is possible that THY-1 interacts directly with gB and that the interaction of THY-1 with gH is indirect and solely due to gH interacting with gB. Alternatively, THY-1 has been shown to bind to integrins, and gB and gH from several herpesviruses interact with integrins; thus, the interaction between THY-1 and gB and gH may be indirect and mediated through integrins.

### THY-1 colocalizes with HCMV gB and gH in infected cells

**Fig 6:**
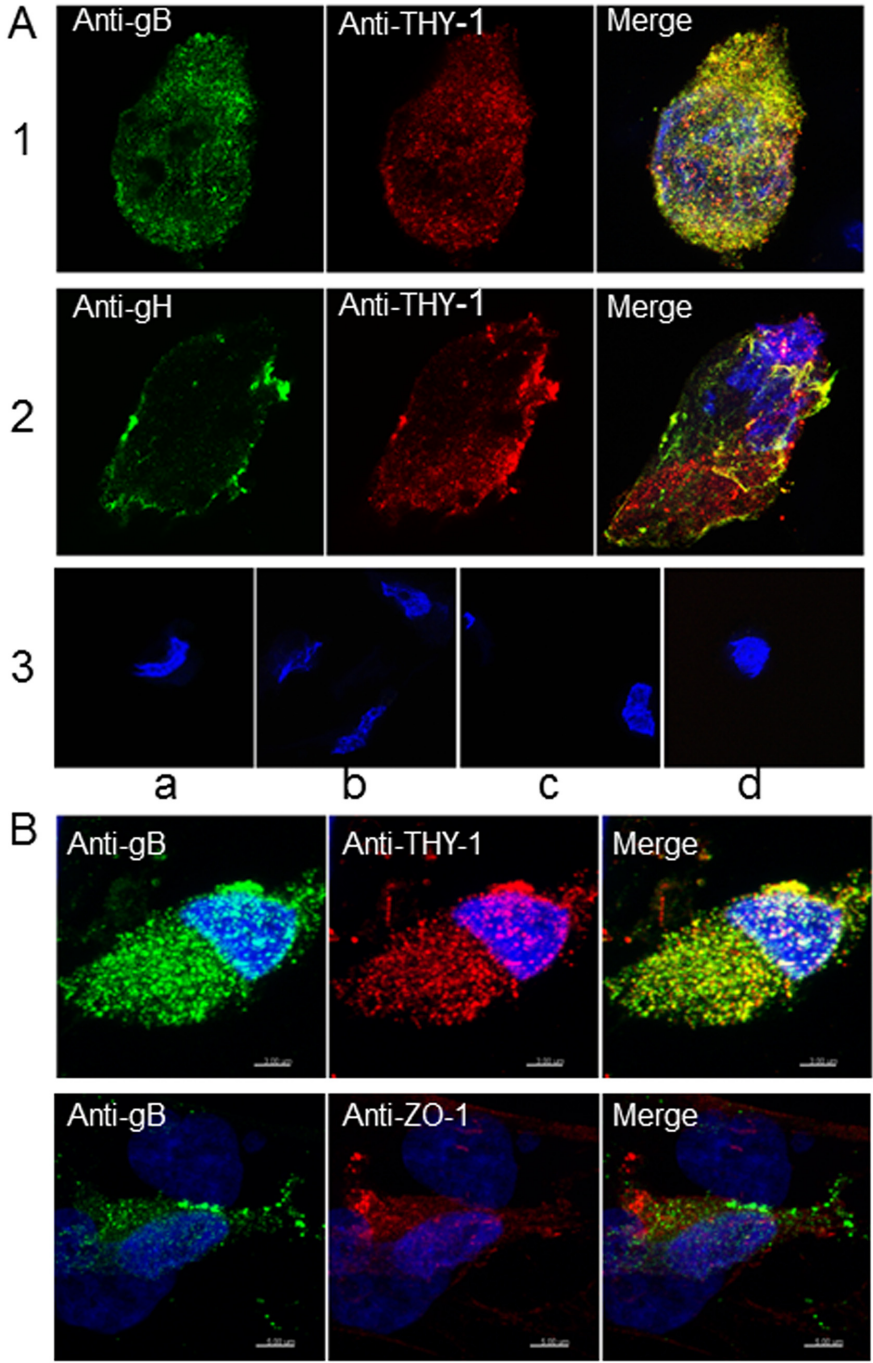
Colocalization of THY-1 with HCMV gB and gH from virus-infected cells by confocal microscopy. (A) MRC-5 cells were infected with HCMV AD169 and live cell (surface) staining was performed with goat anti-THY-1 and mouse anti-HCMV gB antibody (row 1), or mouse anti-HCMV gH antibody (row 2). Anti-goat-Alex 594 (red) and anti-mouse Alexa 488 (green) were used to detect THY-1 and HCMV glycoproteins, respectively. Negative controls for non-specific cross-reactivity and background included HCMV-infected MRC-5 cells stained with goat anti-THY-1 and anti-mouse Alexa 488 (row 3a), anti-goat Alexa 594 with mouse anti-HCMV gB (row 3b), or with mouse anti-HCMV gH (row 3c), or anti-goat Alex 594 and anti-mouse Alexa 488 (row 3d). Nuclei were stained with DAPI (4',6-diamidino-2-phenylindole). (B) Colocalization of gB with THY-1, but not ZO-1, in HCMV AD169-infected HS-578T adenocarcinoma cells by confocal microscopy. Cell staining was performed with mouse anti-HCMV gB antibody, goat anti-THY-1 antibody, or rabbit anti-ZO-1 antibody followed by anti-mouse Alexa 488 (green), anti-goat Alexa 594 (red), or anti-rabbit Alexa 594. Nuclei were stained with DAPI (4',6-diamidino-2-phenylindole). 41% of gB colocalized with THY-1, and 44% of THY-1 colocalized with gB; Pearson’s Correlation Coefficient was 0.27; a coefficient of 1.0 indicates 100% colocalization (top row); 3.2% of gB colocalized with ZO-1, and 2.1% of ZO-1 colocalized with gB; Pearson’s coefficient was -0.1 (bottom row).

**Fig 7:**
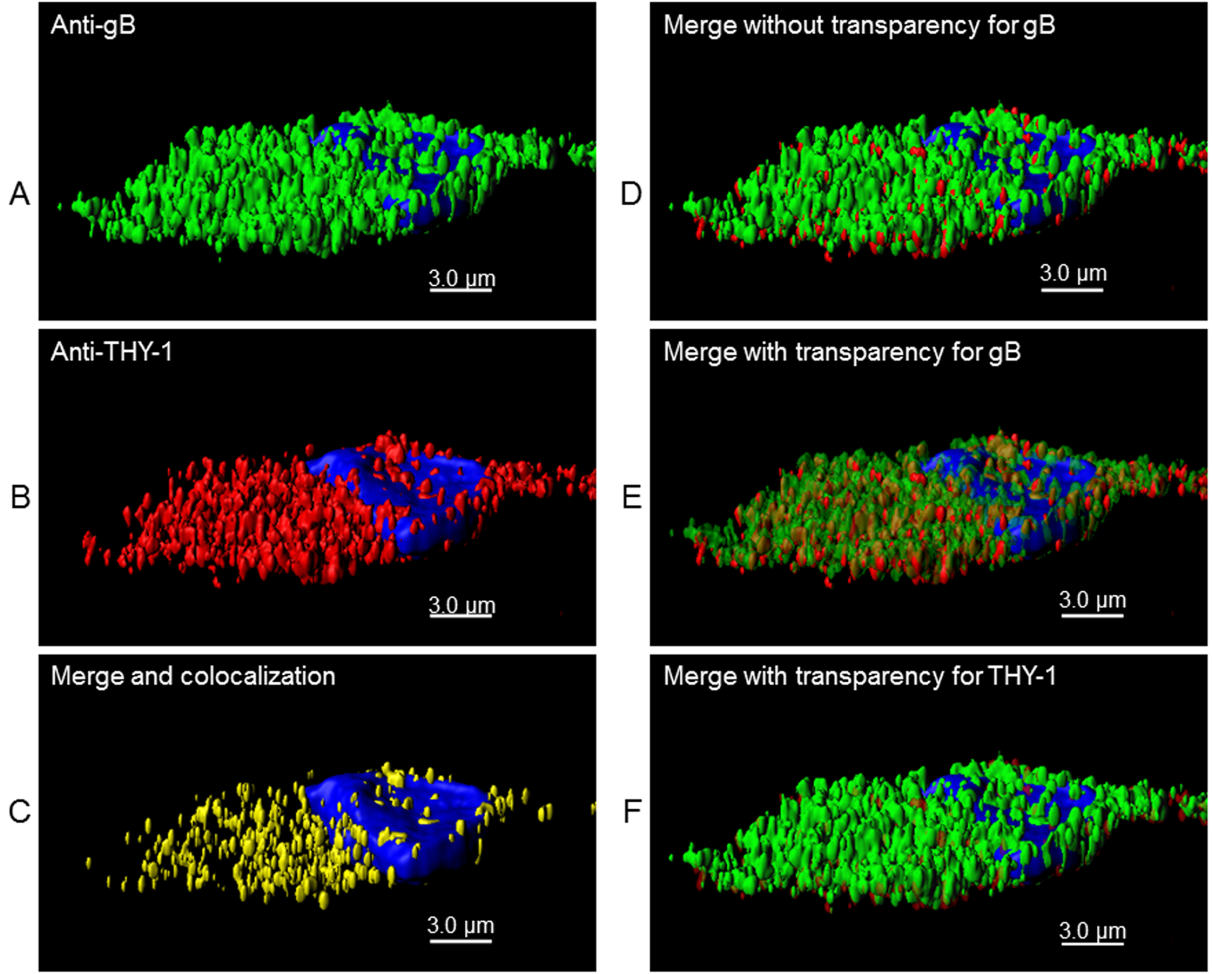
3-D reconstruction of cell membrane showing the orientation of gB relative to THY-1 in HCMV-infected cells by confocal microscopy. Confocal microscopy was performed with HS-578T cells infected with HCMV as described in Fig 6. (A) Surface staining for gB. (B) Surface staining for THY-1. (C) The portion of gB and THY-1 that colocalized is shown. (D) Merge of gB from panel A with THY-1 from panel B. (E) Merge of gB from panel A with THY-1 from panel B in which gB is shown with increased transparency to show the orientation of colocalization of the two proteins on the cell membrane; THY-1 is predominantly underneath gB. (F) Merge of gB from panel A with THY-1 from panel B in which THY-1 is shown with increased transparency. Since THY-1 was predominantly underneath gB, this results in an image similar to panel D. Nuclei were stained with DAPI in each panel.

To further study the possibility of an interaction between THY-1 and the HCMV gB and gH glycoproteins [10], MRC-5 cells were infected with HCMV AD169 (which does not express GFP) and live cell staining was performed with goat anti-THY-1 antibody and mouse monoclonal anti-gB, anti-gH, or isotype control antibody followed by anti-goat and anti-mouse fluorescent antibodies and confocal microscopy. THY-1 colocalized with gB (Pearson Correlation Coefficient 0.88 where 1.0 is 100% colocalization [49] (Fig 6A, row 1) and gH (Pearson Correlation Coefficient 0.84, Fig 6A row 2). Incubation of MRC-5 cells with secondary antibody alone did not give background staining, goat anti-THY-1 did not cross react with secondary anti-mouse fluorescent antibody, and mouse anti-glycoprotein antibodies did not cross react with secondary anti-goat fluorescent antibody (Fig 6A, row 3). In HCMV- infected adenocarcinoma HS-578T cells, gB also colocalized with THY-1 (Figs 6B and S11). As a control, gB did not colocalize with cell surface protein ZO-1 (Fig 6B). Interestingly, confocal microscopy with 3-D reconstruction of the cell surface showed that gB appeared to bind predominantly on top of THY-1 molecules on the plasma membrane (Fig 7). Although gB is conserved among human herpesviruses, HCMV gB (AD169 strain) and VZV gB (Dumas strain) share only 20% amino acid identity and 31% similarity. As an additional control, we co-transfected THY-1 with either HCMV gB or VZV gB, and performed confocal microscopy. HCMV gB colocalized with THY-1 at levels similar to that in infected cells, but VZV gB did not colocalize with THY-1 (S11 Fig).

These results suggest that THY-1 may form a complex with HCMV gB and gH in infected cells. Since glycoproteins gB and gH have been shown to form a complex, it is possibly that THY-1 interacts directly with gB and that the interaction of THY-1 with gH is indirect and solely due to gH interacting with gB. Alternatively, THY-1 has been shown to bind to integrins, and gB and gH from several herpesviruses interact with integrins, thus, the interaction between THY-1 and gB and gH may be indirect and mediated through integrins,

### Down-regulation of THY-1 by siRNA or blocking THY-1 by antibody inhibits HCMV-induced Akt activation

**Fig 8:**
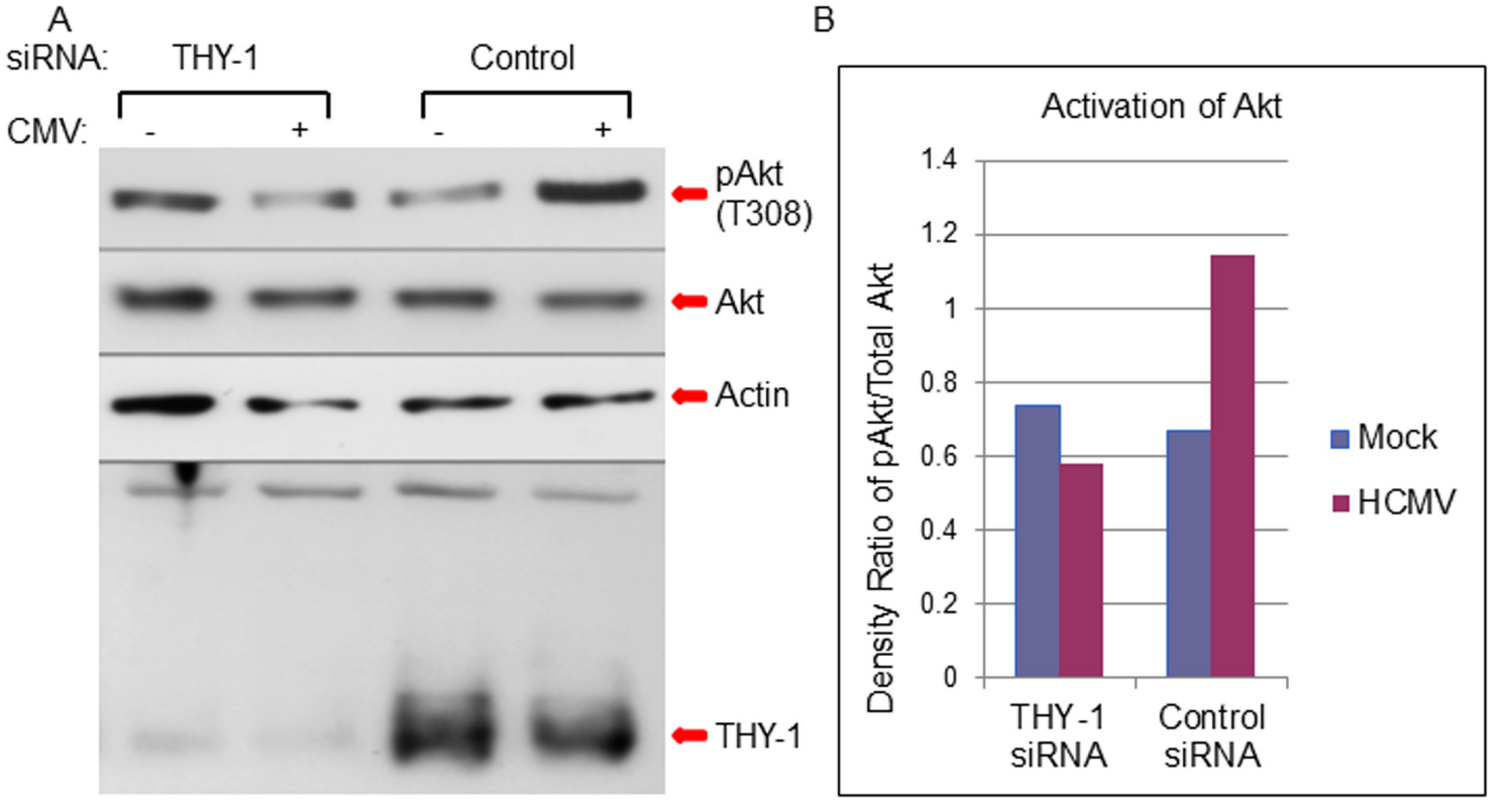
Down-regulation of THY-1 expression blocks HCMV-induced activation of Akt MRC-5 cells were nucleofected with THY-1 specific or control non-targeting siRNAs as described in Fig 1. 48 hrs after transfection, the cells were inoculated with Towne-GFP at 4°C for 60 min for binding and then shifted to 37°C for 15 min to allow synchronized entry. The cells were harvested with lysis buffer (0.1M Tris, 4% SDS and 5% DTT) and proteins were separated on SDS-PAGE gels and immunoblotted sequentially with anti-phosphorylated Akt, anti-actin and anti-THY-1 antibodies. A duplicate membrane was probed with anti-total Akt antibody (A). Densitometry of bands on immunoblots was quantified using ImageJ software (B). A representative experiment is shown from 6 independent experiments performed.

Previous studies have shown THY-1 modulates the phosphatidylinositol 3-kinase (PI3K) signaling pathway [50]. Activation of the PI3K pathway is required for HCMV infection at the entry step [14,20,22]. Therefore, we analyzed the effect of THY-1 on the ability of HCMV to phosphorylate Akt, a downstream molecule in the PI3K pathway. Knock-down of THY-1 expression with specific siRNAs blocked HCMV-induced phosphorylation of Akt at 15 min post-infection and reduced HCMV infectivity within the first 60 min of infection (Figs 8A and 8B and S12) compared with control siRNAs (p = 0.01, 6 independent experiments). These data suggest that HCMV engagement of THY-1 during the initial 15 min of infection contributes to HCMV signaling through the PI3K/Akt pathway.

We then tested whether THY-1 antibody could block HCMV mediated Akt activation during entry. Binding of THY-1 antibody, but not isotype control antibody, to the cell surface inhibited Akt activation within 45 min after the incubation temperature was raised to 37°C to allow for HCMV internalization (S9 Fig).

## Discussion

In spite of progress in the field of virus entry, our understanding of the interaction of viral and cellular proteins required for initiation of HCMV infection is still unclear. This may reflect the large number of HCMV glycoproteins and the ability of the virus to infect a wide variety of cell types. Previous studies of early events in HCMV infection were largely limited to a few cell lines. This imposed limitations for identifying host molecules that are important for infection, since virus entry is cell-type dependent. To address this issue, we studied HCMV infectivity in 54 cell lines with diverse genetic backgrounds. The extensive molecular profiling of each of these cell lines along with bioinformatics analysis allowed us to take an unbiased approach to study virus infection instead of screening for single molecules in isolation. The identification of THY-1 as a putative host determinant for HCMV infection in a large set of 54 cell lines, and the subsequent validation by a series of loss-of-function, gain-of-function, and glycoprotein interaction experiments in both malignant and primary cells strongly suggests that THY-1 has an important role in the initial stage of virus infection. THY-1 is expressed in many cell types both in vivo and in vitro, including epithelial and endothelial cells, smooth muscle cells, placenta, neurons, hepatocytes, and hematopoietic stem cells, the same cells that are susceptible to HCMV infection. Therefore, THY-1 likely facilitates HCMV entry in many cell types. On the other hand, THY-1 may not be required for infection of all cell types; instead, it functions in a cell type dependent manner. Other herpesviruses use different receptors to enter different cell types. HSV uses nectin-1 to enter neurons and HVEM to enter lymphocytes [51]. Some cell lines that express very low levels of THY-1 are still susceptible to HCMV infection, particularly at high m.o.i. or after prolonged virus inoculation. It is likely that HCMV enters cells through different pathways, either by direct fusion at the cell surface or by various endocytic pathways, especially when large amounts of virus are used in vitro. This is similar to the case of Lassa virus infection, in which the impairment of virus glycoprotein mediated entry imposed by deletion of host receptor glycosylated α-dystroglycan can be overcome by using high titer virus (m.o.i > 0.5), resulting in virus entry through an alternate pathway involving heparin sulfate, lysosome-resident protein, and pH-dependent endocytosis [52]. Previous studies have shown for other viruses entry dynamics are highly dependent on the m.o.i. Virus internalization occurs much more rapidly when a high m.o.i. (m.o.i 10) is used, compared to a low m.o.i of 0.01–1 [53,54]. For HCMV, infection at low m.o.i. (≤ 0.01) resulted in different profiles of virus replication and signaling as compared with infection at higher m.o.i (0.1–3.0) [55–57]. In the current study, we used a combination of low m.o.i. (between 0.05–1) and short time for infection (60 minutes followed by inactivation of virus remaining on the cell surface) to focus on the most efficient entry pathway(s). As shown in S5 and S6 Figs, only a fraction of the input virus entered cells within 1 to 2 hours at the low m.o.i. Nonetheless, a low m.o.i. is likely more representative of the virus to cell ratio present during natural infection. Since THY-1 is a major cargo protein of clatherin-independent endocytotic carriers [58], it is possible that THY-1 leads virions into the cells by macropinocytosis. Many viruses down-regulate and internalize their receptors from the cell surface through endocytic pathways [59]. Previous studies have shown that THY-1 is down-regulated in fibroblasts [60,61], as well as in mesenchymal stem cells [62] upon HCMV infection in a manner similar to that of PDGFR-α [63].

THY-1 is known to interact with cell proteins that facilitate HCMV entry. THY-1 engages αVβ3 integrin receptors and recruits paxillin [64], and triggers protein kinase dependent signaling pathways such as PI3K and Src [50,65,66]. THY-1 was important for activation of Akt in virus-infected cells and activation of PI3K –Src pathway has been shown to be required for HCMV entry [14,20,22]. Our findings that THY-1 facilitates an early step of HCMV infection, and that down-regulation of THY-1 by siRNA or blocking THY-1 with antibody inhibits HCMV- induced PI3K-Akt activation within the initial 15–45 min of infection, suggests a pivotal role for THY-1 in the coupling of HCMV entry with host signaling, and supports observations that growth factor receptors (PDGFR- α and EGFR) engage integrin/paxilin pathways during HCMV infection [14,16,22,67]. THY-1 protein is localized in lipid rafts through its GPI anchor. Ligand-mediated clustering of THY-1 in cholesterol-rich microdomains is needed to trigger Src-dependent downstream signaling [68,69]. We hypothesize that THY-1 clustering might be induced by interactions between THY-1 and HCMV gB and/or gH, two molecules that have been reported to contribute to signaling during virus entry [22,70]. This is similar to observations that binding of Group B coxackievirus to its receptor decay-accelerating factor (DAF), a GPI anchoring protein, induces DAF clustering to initiate signaling by Src family kinases [71].

We found that THY-1 interacts with both full length and 55 kD cleavage forms of gB, as well as with gH. Both full length and cleaved forms of gB are present on infected cells and virions [72]. Furthermore, THY-1 colocalizes with gB and gH in HCMV-infected cells. However, it is not clear whether THY-1 interacts with gB or gH directly or indirectly. Several studies have shown that exogenous HCMV gB and gH interact [10,73]. gH/gL have been postulated to function as receptor binding proteins, while gB may act as a fusogen; however, gB also binds to ligands and signaling molecules [8,74]. HCMV gB has been identified as a ligand for putative entry mediators, including integrins, PDGFR-α, and EGFR [14,16,22]. Like THY-1, both EGFR and PDGFR-α have been shown to form a complex with αVβ3 integrin [75,76], and are activated when they oligomerize after binding with ligands [69,77,78]. Therefore, THY-1 may be part of a multimolecular complex mportant for the initial phase of CMV infection and signaling (that includes PDGFR- α, EGFR, integrins, paxillin, and viral glycoproteins). However, it is uncertain how THY-1 fits into this complex and the exact form and timing of interaction(s) between THY-1, gB, and gH are unclear. Previous studies of HSV showed recruitment of other viral and host molecules to a complex after gD binds to its receptor. Since THY-1 interacts with several other cellular proteins, including integrins and is important in multiple signaling pathways, it is likely that THY-1 facilitates HCMV infection at an early stage as an entry mediator, rather than a receptor for a viral glycoprotein(s).

HCMV disseminates in leukocytes throughout the body after infecting mucosal epithelial cells, and induces production of inflammatory cytokines and increases permeability of the endothelium. This process is dependent on activation of the PI3K signaling pathway, which promotes extravasation of leukocytes into tissues [67,79,80]. Binding of THY-1 induces vascular permeability and regulates the extravasation of leukocytes during inflammation [81]. THY-1 is expressed in many types of cells that can be productively infected by HCMV as well as CD34^+^/CD38^-^ stem cells, a putative cellular reservoir for latent infection [62,82]. Therefore, THY-1 may have a central role in mediating HCMV infectivity, coupling integrin/paxillin and leukocyte extravasation signaling, and linking the process of viral entry with signaling modulation of host cells that leads to the virus replication.

## Materials and Methods

### Cells, viruses and reagents

Human diploid fibroblast (MRC-5), retinal pigmented epithelial (ARPE-19) cells, and CV1/ EBNA-1 cells were acquired from the American Type Culture Collection (ATCC, Manassas, VA), and maintained in Minimum Essential Medium, F12 medium, or Dulbecco’s Modified Eagle Medium, respectively, with 10% FBS.

HCMV antibodies used were monoclonal anti-gB (Virusys, Taneytown, MD), monoclonal anti-gH (US Biological, Swampscott, MA), rabbit anti-gH antibodies (from Teresa Compton, University of Wisconsin and. David Johnson, Oregon Health & Science University), and mouse anti-ICP 8 antibody (Novus, Littleton, CO). Monoclonal antibodies used to detect THY-1 or isotype control antibody were purchased from Novus (Littleton, CO), Millipore (Billerica, MA) and BioLegend (San Diego, CA). Polyclonal goat anti-THY-1 or rabbit anti-THY-1 were obtained from Novus and GeneTex (Irvine, CA). Monoclonal antibodies for total and phosphorylated Akt were purchased from Cell Signaling Technology (Boston, MA). Expression plasmids encoding HCMV gB and gH were kindly provided by Teresa Compton [21]. pCMV-THY-1 was purchased from Open Biosystems (Huntsville, AL), and pCR2.1-GAPDH was a gift from Dr. Helene Rosenberg (NIAID, NIH). Plasmid expressing VZV full length gB was constructed by PCR amplification of gB ORF from the Oka strain of VZV, cloned into pcDNA3.1 vector with a C-terminal V5 epitope tag, and verified by sequencing.

BAC DNAs for epithelial/endothelial tropic HCMV strains BADrUl131-GFP and TB40E-GFP (kindly provided by Thomas Shenk, Princeton University, NJ; TB40E-GFP is also referred to as GS1783TB40-GFP) [37] were electroporated into ARPE-19 cells and the resulting viruses were propagated in ARPE-19 cells. Towne-GFP and AD169, both fibroblast topic HCMV strains, were propagated in MRC-5 cells. Cell culture supernatants from virus-infected cells were centrifuged at 2000 x g for 30 min at 4°C, and the clarified supernatants were used as virus stocks or further partially purified by centrifugation through a 20% sucrose or sorbitol cushion with a JA25 rotor at 35000 x g at 4°C for 60 min and resuspended in growth medium [83,84]. GFP expressing adenovirus (adenovirus-GFP) and herpes simplex virus 2 (HSV-2-GFP) were used as controls [85,86].

### Screening for host molecules important for HCMV infection

54 cell lines from the NCI-Frederick Tumor Cell Line Repository were purchased through Charles River Laboratories (Frederick, Maryland) and grown in RPMI-1640 medium with 10% FBS [34]. Cells with less than 20 passages after receipt were used in the study. Screening was performed based on previously published methods that have been used to identify other proteins important for the early stage of virus infection [29,30,32,87]. Briefly, cells were infected with GFP-expressing HCMV for 2–3 days, and susceptibility to HCMV was determined by FACS analysis based on GFP positivity, and normalized against infectivity for MRC-5 (for fibroblast tropic virus) or ARPE-19 (for epithelial and endothelial tropic viruses) cells. Bioinformatic analyses to determine correlations between HCMV infectivity and expression of each cellular gene were performed using the COMPARE algorithm and further detailed using MAPP software as described previously [30,38].

### Virus entry assay

HCMV was added to cells on ice for 60 min for virus binding. The temperature was then shifted to 37°C for 60 min to allow virus entry. The cells were then treated with low pH citrate buffer (sodium citrate 40 mM, potassium chloride 10 mM, sodium chloride 135 mM, pH 3.2) at room temperature for 3 min to inactivate any virus that had not yet internalized, washed twice, and cultured for either 6 hrs to detect viral encoded RNAs or 3 days to quantify GFP positivity in cells infected with HCMV expressing GFP. A low m.o.i. (0.05–1) was used for most virus entry experiments since this is likely what occurs during natural infection; a high m.o.i (with a high percentage of cells infected) has been shown to result in different kinetics of entry than a low m.o.i. [53,55,88]. GFP positivity was determined by FACS analysis using a BD FACSCalibur and data was analyzed with FlowJo software (Tree Star, Ashland, OR).

### Identification of protein-protein interaction by pull-down assay and mass spectrometry

MRC-5 cells infected with HCMV at an m.o.i. of 5 for 3 days or uninfected control cells were lysed in buffer (25 mM Tris, 15 mM NaCl, 0.1% NP40 with or without 5mM EDTA) as described previously [46]. Immunoprecipitation with anti-THY-1 antibody and protein A-Sepharose (Sigma-Aldrich, St. Louis, MO) was carried out at 4°C overnight. After extensive washing, proteins were separated in SDS-PAGE gels under reducing conditions and visualized by Coomassie Blue or silver staining. Specific bands were excised and subjected to mass spectrometry for protein identification (Research Technologies Branch, NIAID, NIH).

### HCMV protein binding assays

Soluble THY-1-His protein, or VZV gE-His control protein, was bound to Ni-NTA or Talon columns at 4°C for 2 hr followed by extensive washing with PBS. HCMV-infected or uninfected cell lysates were centrifuged at 2000 x g at 4°C for 30 min, and the supernatant was added to columns and incubated at 4°C overnight with rotation. Columns were washed with PBS using a peristaltic pump, and eluted with 250mM imidazole. Samples were concentrated by centrifugation at 1600 x g in an Amicon Ultra centrifugal filter unit (3000 MWCO), separated in SDS-PAGE gels, transferred to nitrocellulose membrane, and immunoblotted with antibodies.

### RNA extraction and RT-qPCR

Total RNA was extracted using an RNeasy Mini Kit (Qiagen, Valencia, CA) following the manufacturer’s instructions. To eliminate DNA contamination, RNA was treated with DNase I (Roche Applied Science, Indianapolis, IN) and purified a second time with an RNeasy Mini Kit. Quantitative real-time RT-PCR was performed using One-step RT-PCR Master mix reagent (Applied Biosystems, Carlsbad, CA) with a 7500 Real Time PCR machine. Primers and probes for detection of HCMV immediate-early gene UL123 and late gene UL55 were described previously (Boeckh et al., 2004). Primers (5’- GTTAGGCTGGTCACCTTCTG, 5’- GAGATCCCAGAACCATGAACC) and probe (5’- AGACTGTTAGCAGGAGAGCGATGC) for THY-1 were located in exon 1. Primers and probe for GAPDH were purchased from Applied Biosystems (Carlsbad, CA). Serial dilutions of HCMV Bac DNA, THY-1 plasmid, or human GAPDH plasmid were used to generate standard curves, and copy numbers of THY-1 and HCMV RNAs were normalized to copy numbers of human GAPDH amplified from the same wells. DNA contamination was monitored by performing PCR amplification without reverse transcriptase.

### Knockdown of host gene expression by siRNA

THY-1 specific siRNA SmartPools (M-015337-00) and non-specific control pools (Duplex-13), THY-1 single siRNA oligos with targeting sequences CAACUUCACCAGCAAAUAC (THY-1-02) and GGACUGCCGCCAUGAGAAU (THY-1-04), and non-targeting single oligo #4 were obtained from Dharmacon (Lafayette, CO). Cells were transfected with siRNAs (125 pmol per 2 x 10^6^ cells) using nucleofection (Amaxa, Gaithersburg, MD) for 48 hr before infection or harvesting.

### Cloning, expression, and purification of soluble THY-1 protein

DNA corresponding to THY-1 amino acids 20–130 with a C-terminal (His)6 tag was amplified by PCR from plasmid pCMV-THY-1 (using primers 5’-CAGAAGGTGACCAGCC and 5’-GCTCAGAGACAAACTGGTCAAG, and cloned into pDC409 [89]. The THY-1 insert in the resulting plasmid, pDC409-THY-1(20–130)-His, was completely sequenced. THY-1-His soluble protein was expressed in CV1/EBNA 1 cells and purified with a Ni-NTA column (Invitrogen, Grand Island, NY) or Talon resin (Clontech, Mountain View, CA), eluted with 250 mM imidazole, dialyzed against PBS at 4°C overnight, and concentrated with an Amicon Ultra centrifugal filter unit (3000 MWCO) (Millipore, Billerica, MA). Filtrates derived from this filter unit with the same buffer composition, but lacking THY-1 protein, were used as a negative control for experiments. Soluble varicella-zoster virus gE with a C-terminal (His)6 tag, gE-His [46], was used as an additional control in soluble THY-1-His protein experiments.

### Immunofluorescence microscopy

Cells were fixed in methanol/acetone (1:1) at -20°C for 5 min. After washing in PBS, blocking buffer (4% BSA and 10% normal goat serum in PBS) was added for 1 hr before incubation with mouse monoclonal anti-HCMV gB or gH, and goat anti-THY-1 for 60 min on ice, followed by anti-mouse Alexa-488 or anti-goat Alexa-594 (Invitrogen, Grand Island, NY) on ice for 60 min. For cell surface staining, live cells were treated with blocking buffer on ice for 30 min before incubation with primary and secondary antibodies. After antibody staining, cells were fixed with 2% paraformaldehyde, and mounted with DAPI-Fluoromount-G (Southern Biotech, Birmingham, AL). Confocal imaging was performed with a Leica SP5 X-WLL microscope.

## Supporting Information

S1 Fig (A) Transfection of PDGFR-α specific siRNA impairs HCMV infectivity. MRC-5 cells were nucleofected with siRNA to PDGFR-α (M-003162, Dharmarcom, Lafayette, CO) or negative control siRNA (50 pmol/million cells), and infected with TB40E-GFP CMV (m.o.i. 0.5) at 48 hours post-transfection. FACS analysis of infectivity was performed at day 3 post-infection. (B) SNB-19, but not SF-539 cells, express THY-1 protein on the cell surface. Live cells were stained with THY-1 monoclonal antibody 5E10 or isotype control antibody on ice, followed by anti-mouse antibody conjugated with Alexa488, fixed with 2% paraformaldehyde, and analyzed by FACS.
(TIF)

S2 Fig HS-578T and SNB-19 cells support HCMV productive infection. (A) HS-578T cells were infected with Towne-GFP CMV at m.o.i. <0.01. Cell-to-cell spread of progeny virus was observed from day 5 post-infection. (B) SNB-19 cells were infected with Towne-GFP CMV, and 10 days later progeny virus in the cell supernatant was passaged onto MRC-5 cells.
(TIF)

S3 Fig Soluble THY-1 protein blocks HCMV entry in a dose-dependent manner. (A) Percent infectivity of HCMV in HS-578T (adenocarcinoma) cells in the presence of soluble THY-1 protein or control soluble VZV gE used to derive the percentage of relative infectivity shown in Fig 2A. Error bars indicate standard errors. (B) Corresponding raw data from the FACS analysis. Only one set of the triplicates was shown.
(TIF)

S4 Fig Soluble THY-1 protein specifically blocks HCMV infection, but has no effect on HSV-2 or adenovirus infection. (Top) Percent infectivity of HCMV used to derive the percentage of relative infectivity shown in Fig 2B (MRC-5 cells) and Fig 2C and 2D (HS-578T). Error bars indicate standard errors. (Bottom) Corresponding raw data from the FACS analysis.
(TIF)

S5 Fig HCMV entry kinetics in HS-578T, SNB-19 and MRC-5 cells. TB40E-GFP CMV was allowed to bind to HS-578T (A), SNB-19 (B) or MRC-5 (C) cells on ice for 45 min. Virus entry was controlled by raising the temperature to 37°C for the indicated time and terminated by washing the cells in low pH citrate buffer (pH 3.2) for 3 min. Infectivity was analyzed by FACS 3 days after infection. (m.o.i for MRC-5 was 1.0; m.o.i for HS-578T and SNB-19 was 2.0, based on titer obtained on MRC-5 cells). Error bars indicate standard errors.
(TIF)

S6 Fig Soluble THY-1 protein blocks HCMV infection more efficiently during the first hour of infection than after one hour. HS-578T cells were infected with Towne-GFP at m.o.i 4.0 (based on MRC-5 titer) in the presence of soluble THY-1 protein (5 μg/ml) or control soluble VZV gE that has the same number of “His units” determined by ELISA, as described in Fig 2A. After virus binding on ice, the temperature was raised to 37°C for 60 min. The cells were then treated with low pH inactivation or left untreated. At 6 days after infection, infectivity was measured as the percentage of GFP-positive cells by FACS. Error bars indicate standard errors.
(TIF)

S7 Fig THY-1 antibody blocks HCMV entry in a dose-dependent manner. (A) Different amounts of anti-THY-1 antibody (5E10) or isotype control antibody were added to HS-578T cells for 60 min on ice to allow binding to the cell surface. The unbound antibody was then washed off and the cells were infected with HCMV as described above in Fig 3 to allow entry for 60 min. At 3 days after infection, the percentage of GFP-positive cells was determined by FACS. Error bars indicate standard errors. (B) Corresponding raw data from the FACS analysis. Only one set of the triplicates is shown.
(TIF)

S8 Fig THY-1 antibody also blocks HCMV infection in MRC-5 cells. (A) Percent infectivity of HCMV in MRC-5 cells used to derive the percentage of relative infectivity shown in Fig 3E. Error bars indicate standard errors. (B) Corresponding raw data from the FACS analysis. Only one set of the triplicates is shown.
(TIF)

S9 Fig Blocking cell surface THY-1 with specific antibody inhibits HCMV-induced activation of Akt during entry. Anti-THY-1 antibody (5E10) or isotype control antibody was bound to the cell surface of MRC-5 cells on ice for 60 min. Towne-GFP virus was then added at an m.o.i. of 5.0 on ice for a 60 min. The temperature was increased to 37°C to allow virus entry. At the end of the indicated time, the cells were treated with low pH wash and lysed for Western blot as described in Fig 8 (A). The density of specific bands was quantified using Image J software (B).
(TIF)

S10 Fig THY-1 specific siRNA effectively knocks down total THY-1 protein, but only partially down-regulates cell surface THY-1 protein. (A) HS-578T adenocarcinoma cells were nucleofected with THY-1 specific siRNA or negative control siRNA, cell lysates were harvested 48 hrs later, separated on SDS-PAGE gels, and probed with anti-THY-1 antibody. (B) HS-578T adenocarcinoma cells were nucleofected with THY-1 specific siRNA or negative control siRNA and 48 hrs later cell surface expression of THY-1 was measured by immunofluorescent staining with THY-1 antibody 5E10.
(TIF)

S11 Fig HCMV gB, but not VZV gB, colocalizes with THY-1 in transfected cells. HS-578T adenocarcinoma cells were infected with HCMV AD169. 293T cells were cotransfected with the pTHY-1 expression plasmid and either a plasmid expressing HCMV gB or VZV gB. 48 hours later, the cells were stained with goat anti-THY-1, mouse anti-HCMV gB or mouse anti-VZV gB antibody followed by secondary antibodies (anti-goat Alexa 594, red, for THY-1 and anti-mouse Alexa 488, green, for gBs), and confocal microscopy and colocalization analysis were performed as described in Fig 6. (A) Z-stack images stained for HCMV and VZV gB (green) and THY-1 (red). HCMV gB colocalizes with THY-1 (yellow) in both infected and transfected cells, but VZV gB does not colocalize with THY-1. (B) Statistical analysis of colocalization.
(TIF)

S12 Fig Down-regulation of THY-1 expression by siRNA blocks HCMV-induced activation of Akt and HCMV infection. MRC-5 primary cells were nucleofected with THY-1 siRNA or control siRNA as described in Fig 8. 48 hrs after transfection, the cells were inoculated with Towne-GFP at 4°C for 60 min to allow binding and then shifted to 37°C for 15 min to allow synchronized entry. Cell lysates were prepared for immunoblots with anti-Akt and anti-phosphorylated Akt antibodies. (A) Densitometry of bands on immunoblots was quantified using ImageJ software, and the mean of the ratio of the density of pAkt/total Akt bands from six independent experiments is shown. Error bars indicate standard errors. (B) HCMV infectivity in siRNA treated MRC-5 cells. Cells were nucleofected with THY-1 siRNA or control siRNA and 48 hr later the cells were infected with CMV Towne-GFP on ice for 60 min followed by 37°C for 60 min to allow virus entry. Infectivity was scored by FACS at day 3 post-infection. The percent of relative infectivity (compared with the negative control set at 100%) from six independent experiments is shown. Error bars indicate standard errors.
(TIF)

S1 Table Origins of 54 human cell lines. Names and origins of the 54 cell lines used in the initial HCMV infectivity screen. The cell lines were purchased from Charles River Laboratories (Frederick, Maryland), and maintained in PRMI medium with 10% FBS. The cell line numbers listed are corresponding to the numbers shown in Fig 1.
(DOCX)

S2 Table HCMV infectivity of 54 cell lines. Representative raw FACS data of HCMV susceptibility in 54 cell lines. Only one set of the multiple infections of 10 cell lines is shown.
(DOCX)

S3 Table HCMV encoded proteins identified by mass spectrometry. HCMV encoded proteins identified by mass spectrometry from the pull-down assay by anti-THY-1 antibody. Only those with ΣCoverage score greater than 2.0 were shown (ΣCoverage score measures the number of peptides recovered for each protein and the protein coverage. The threshold for statistical significance in the bioinformatics analysis was set at 0.5).
(DOCX)
