# Supplementary figures and images for: THY-1 Cell Surface Antigen (CD90) Has an Important Role in the Initial Stage of Human Cytomegalovirus Infection
Source: PLoS Pathog. 2015 Jul 6;11(7):e1004999. doi: 10.1371/journal.ppat.1004999 (PMC4492587; doi:10.1371/journal.ppat.1004999)

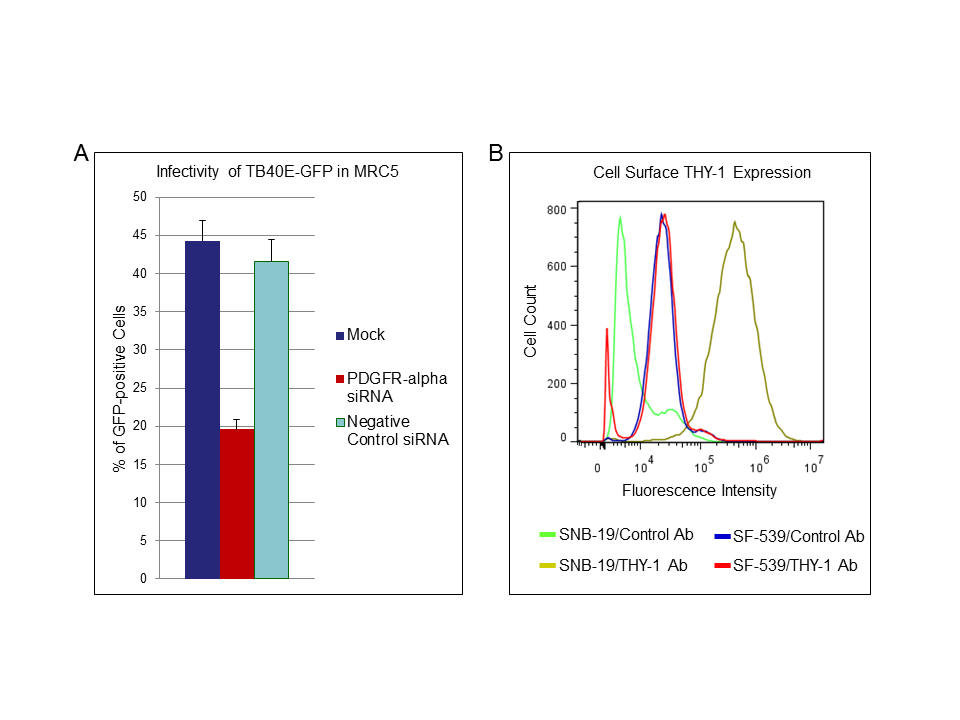

Supplement: S1 Fig — MRC-5 cells were nucleofected with siRNA to PDGFR-α (M-003162, Dharmarcom, Lafayette, CO) or negative control siRNA (50 pmol/million cells), and infected with TB40E-GFP CMV (m.o.i. 0.5) at 48 hours post-transfection. FACS analysis of infectivity was performed at day 3 post-infection. (B) SNB-19, but not SF-539 cells, express THY-1 protein on the cell surface. Live cells were stained with THY-1 monoclonal antibody 5E10 or isotype control antibody on ice, followed by anti-mouse antibody conjugated with Alexa488, fixed with 2% paraformaldehyde, and analyzed by FACS. (TIF) [file ppat.1004999.s001.tif]

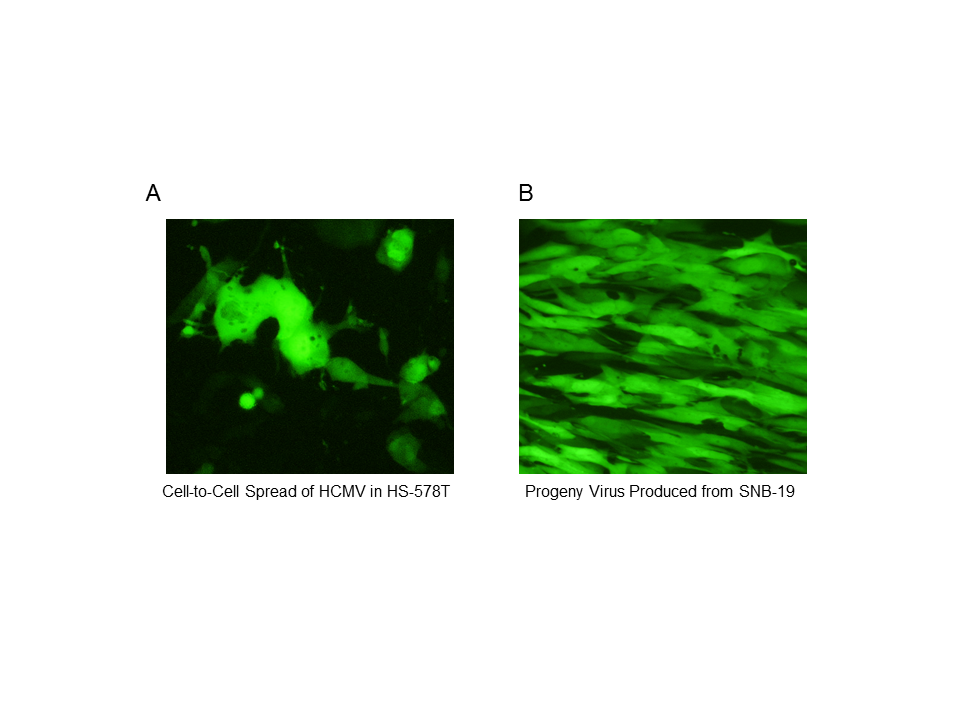

Supplement: S2 Fig — (A) HS-578T cells were infected with Towne-GFP CMV at m.o.i. <0.01. Cell-to-cell spread of progeny virus was observed from day 5 post-infection. (B) SNB-19 cells were infected with Towne-GFP CMV, and 10 days later progeny virus in the cell supernatant was passaged onto MRC-5 cells. (TIF) [file ppat.1004999.s002.tif]

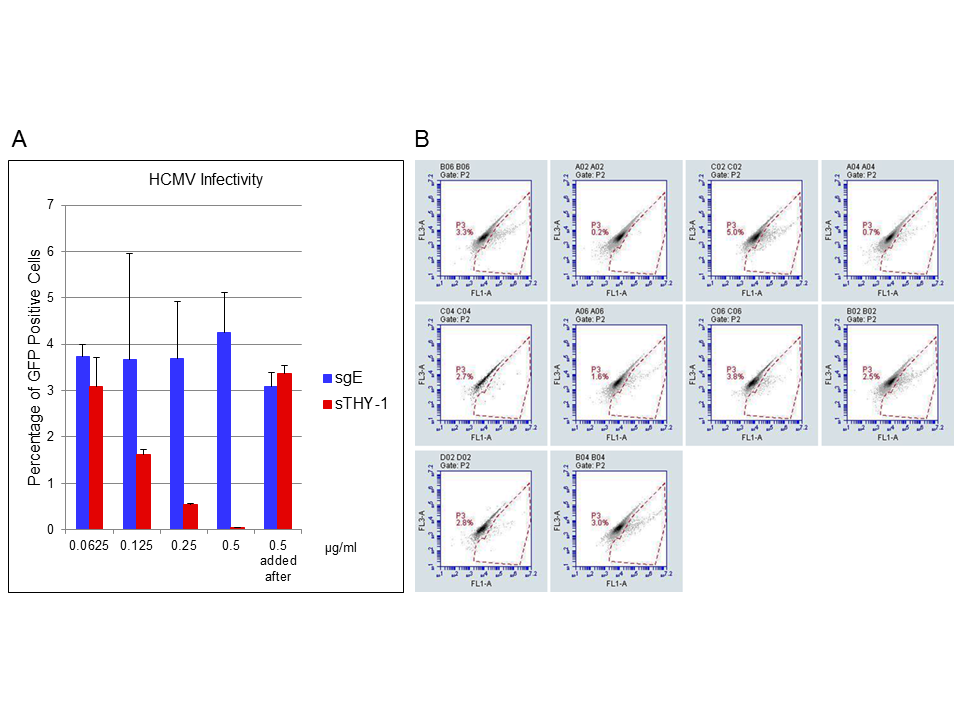

Supplement: S3 Fig — (A) Percent infectivity of HCMV in HS-578T (adenocarcinoma) cells in the presence of soluble THY-1 protein or control soluble VZV gE used to derive the percentage of relative infectivity shown in Fig 2A. Error bars indicate standard errors. (B) Corresponding raw data from the FACS analysis. Only one set of the triplicates was shown. (TIF) [file ppat.1004999.s003.tif]

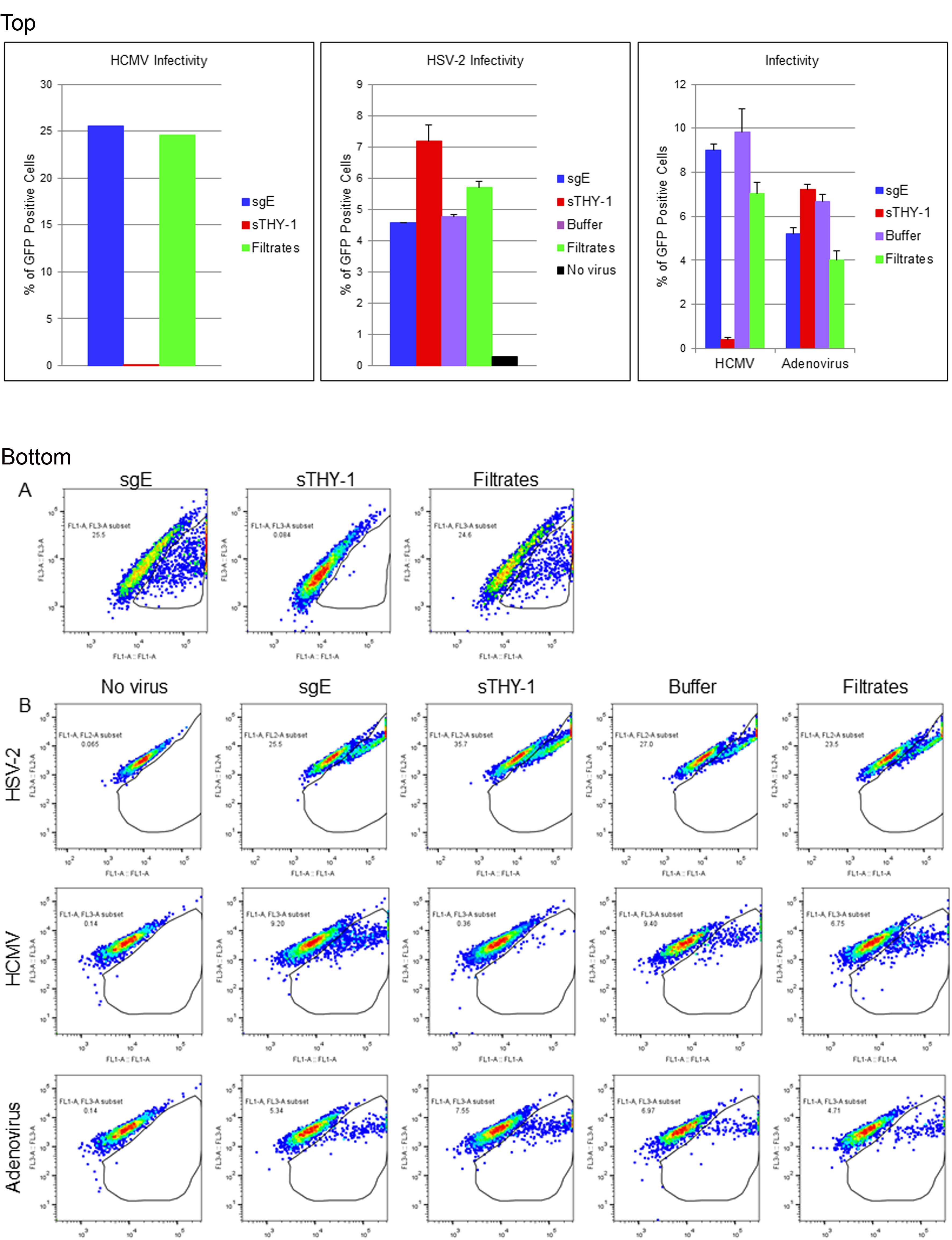

Supplement: S4 Fig — (Top) Percent infectivity of HCMV used to derive the percentage of relative infectivity shown in Fig 2B (MRC-5 cells) and Fig 2C and 2D (HS-578T). Error bars indicate standard errors. (Bottom) Corresponding raw data from the FACS analysis. (TIF) [file ppat.1004999.s004.tif]

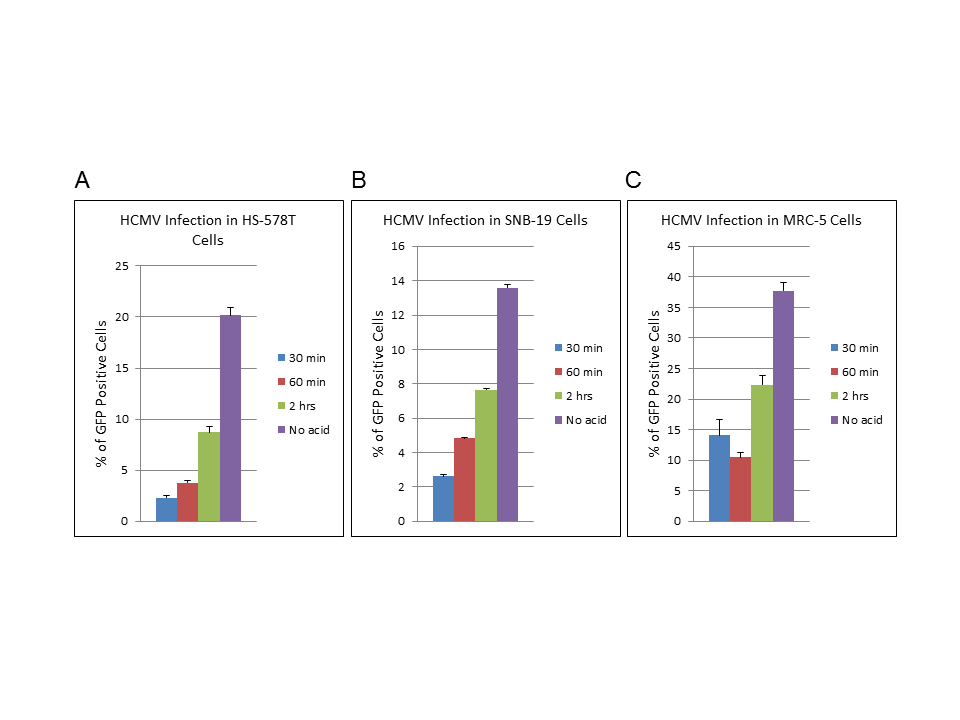

Supplement: S5 Fig — TB40E-GFP CMV was allowed to bind to HS-578T (A), SNB-19 (B) or MRC-5 (C) cells on ice for 45 min. Virus entry was controlled by raising the temperature to 37°C for the indicated time and terminated by washing the cells in low pH citrate buffer (pH 3.2) for 3 min. Infectivity was analyzed by FACS 3 days after infection. (m.o.i for MRC-5 was 1.0; m.o.i for HS-578T and SNB-19 was 2.0, based on titer obtained on MRC-5 cells). Error bars indicate standard errors. (TIF) [file ppat.1004999.s005.tif]

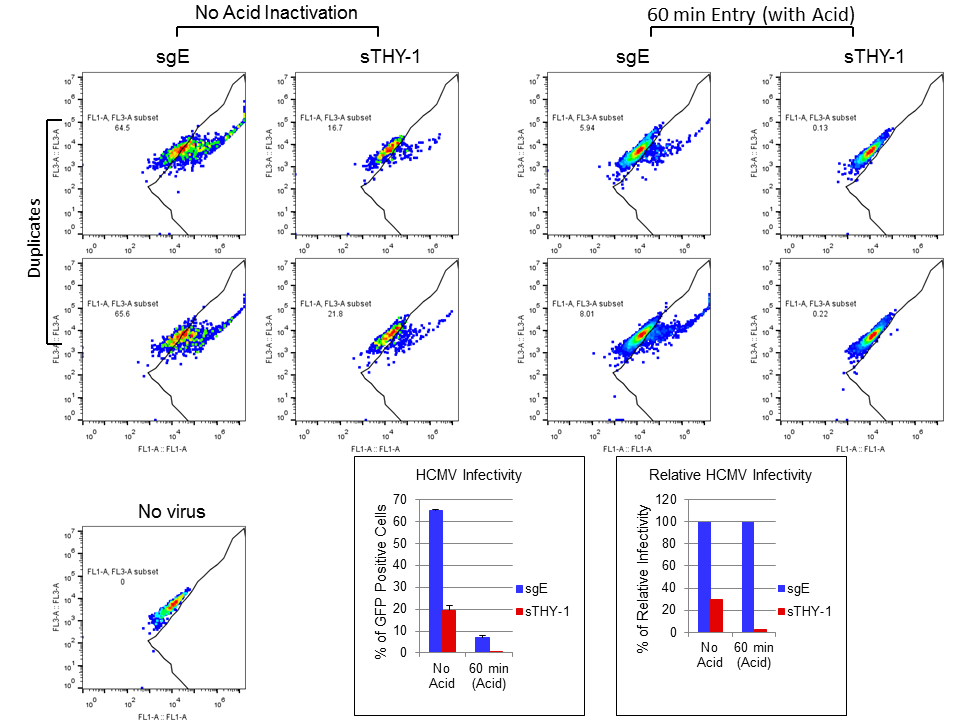

Supplement: S6 Fig — HS-578T cells were infected with Towne-GFP at m.o.i 4.0 (based on MRC-5 titer) in the presence of soluble THY-1 protein (5 μg/ml) or control soluble VZV gE that has the same number of “His units” determined by ELISA, as described in Fig 2A. After virus binding on ice, the temperature was raised to 37°C for 60 min. The cells were then treated with low pH inactivation or left untreated. At 6 days after infection, infectivity was measured as the percentage of GFP-positive cells by FACS. Error bars indicate standard errors. (TIF) [file ppat.1004999.s006.tif]

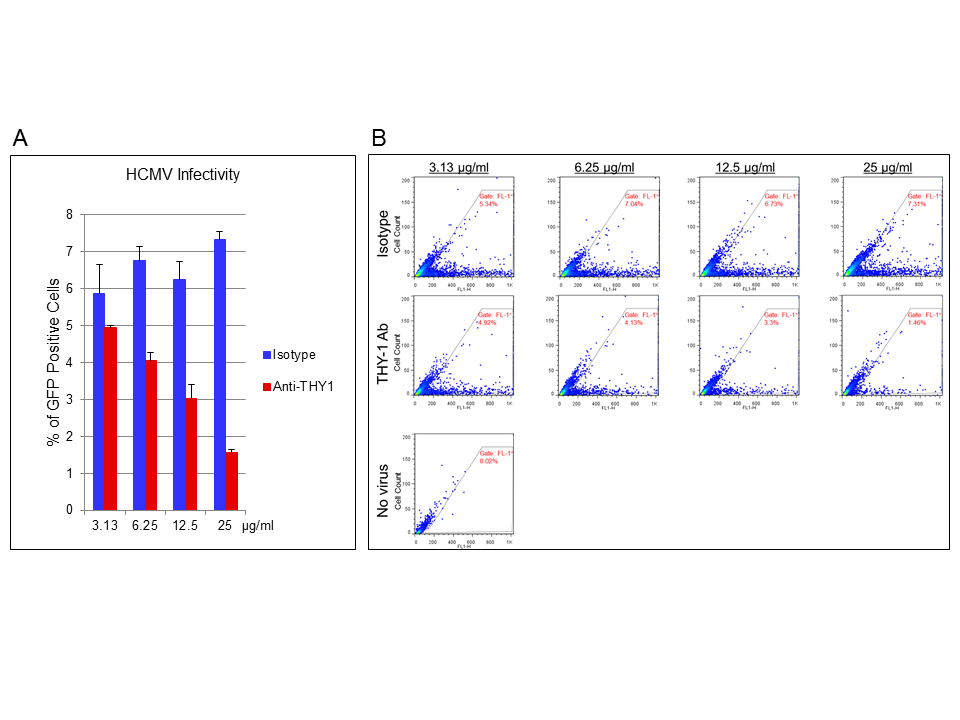

Supplement: S7 Fig — (A) Different amounts of anti-THY-1 antibody (5E10) or isotype control antibody were added to HS-578T cells for 60 min on ice to allow binding to the cell surface. The unbound antibody was then washed off and the cells were infected with HCMV as described above in Fig 3 to allow entry for 60 min. At 3 days after infection, the percentage of GFP-positive cells was determined by FACS. Error bars indicate standard errors. (B) Corresponding raw data from the FACS analysis. Only one set of the triplicates is shown. (TIF) [file ppat.1004999.s007.tif]

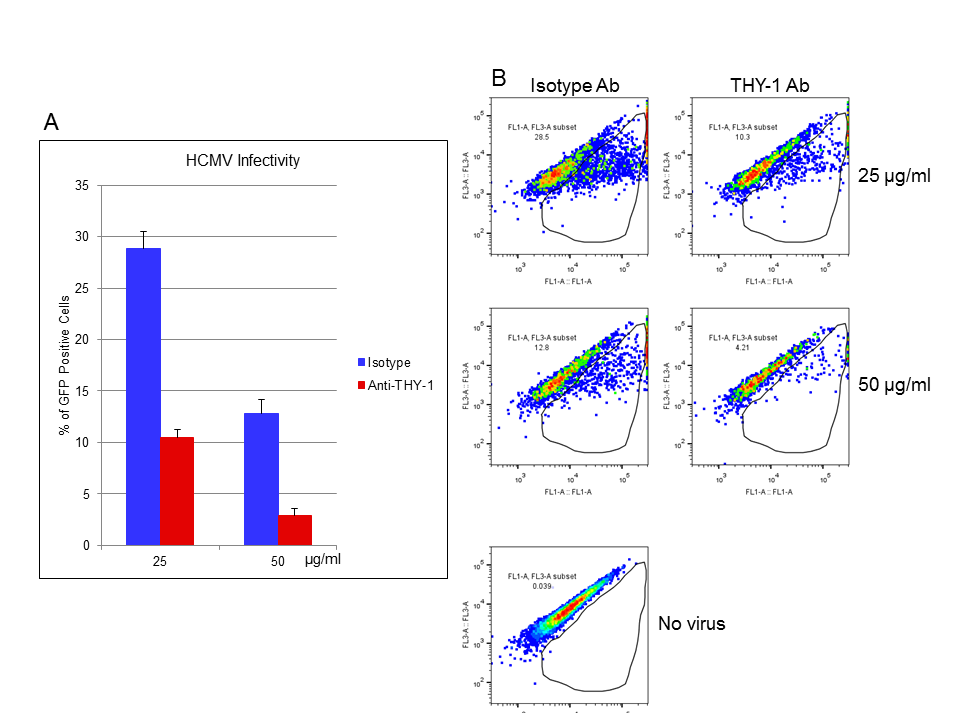

Supplement: S8 Fig — (A) Percent infectivity of HCMV in MRC-5 cells used to derive the percentage of relative infectivity shown in Fig 3E. Error bars indicate standard errors. (B) Corresponding raw data from the FACS analysis. Only one set of the triplicates is shown. (TIF) [file ppat.1004999.s008.tif]

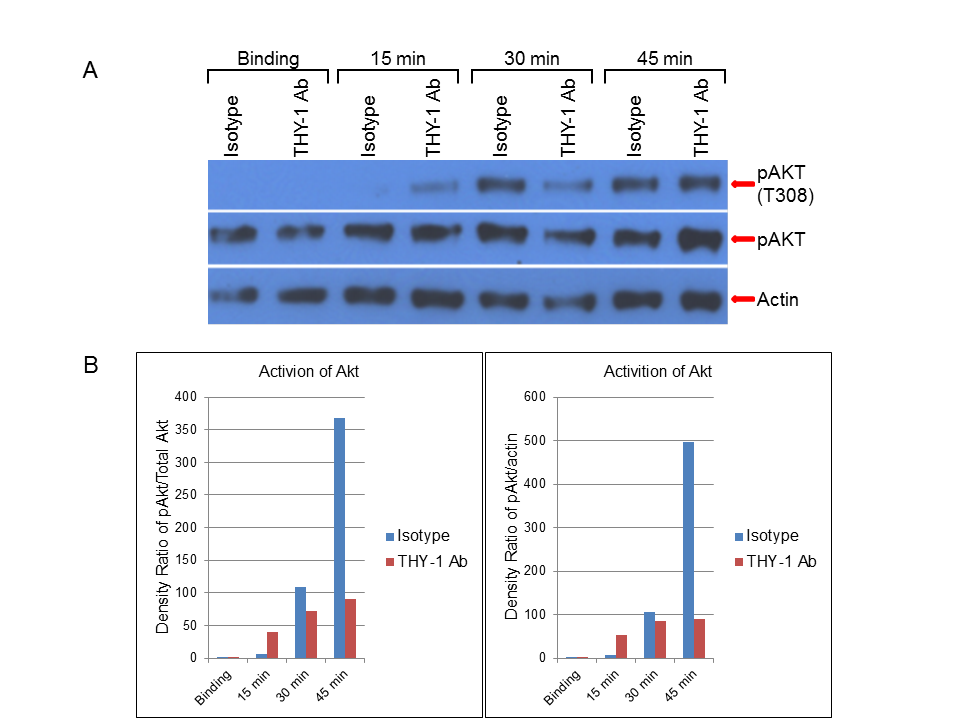

Supplement: S9 Fig — Anti-THY-1 antibody (5E10) or isotype control antibody was bound to the cell surface of MRC-5 cells on ice for 60 min. Towne-GFP virus was then added at an m.o.i. of 5.0 on ice for a 60 min. The temperature was increased to 37°C to allow virus entry. At the end of the indicated time, the cells were treated with low pH wash and lysed for Western blot as described in Fig 8 (A). The density of specific bands was quantified using Image J software (B). (TIF) [file ppat.1004999.s009.tif]

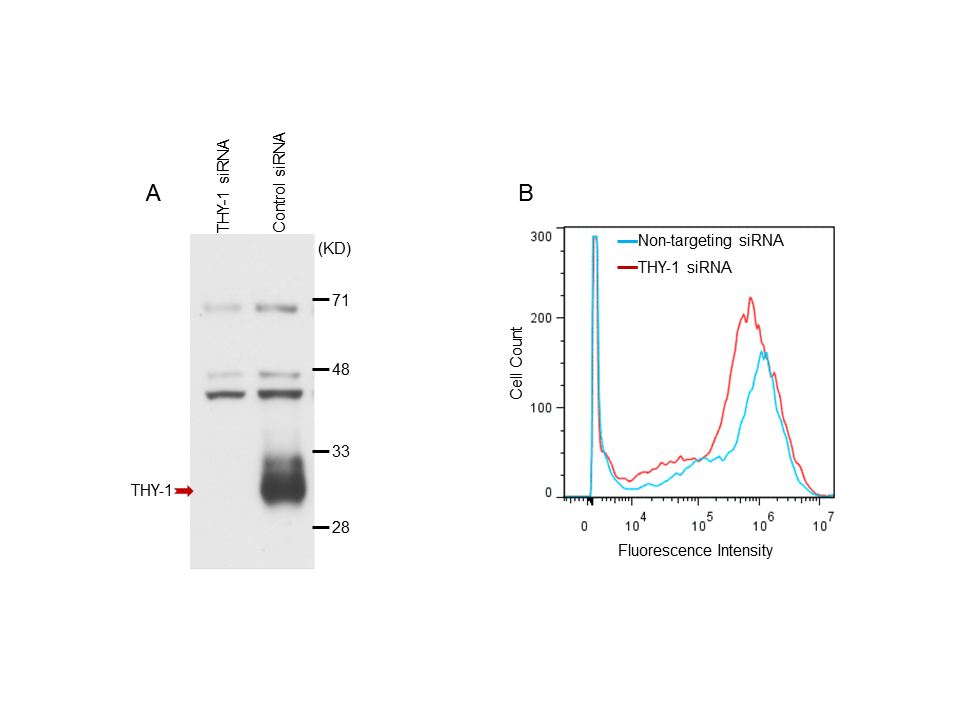

Supplement: S10 Fig — (A) HS-578T adenocarcinoma cells were nucleofected with THY-1 specific siRNA or negative control siRNA, cell lysates were harvested 48 hrs later, separated on SDS-PAGE gels, and probed with anti-THY-1 antibody. (B) HS-578T adenocarcinoma cells were nucleofected with THY-1 specific siRNA or negative control siRNA and 48 hrs later cell surface expression of THY-1 was measured by immunofluorescent staining with THY-1 antibody 5E10. (TIF) [file ppat.1004999.s010.tif]

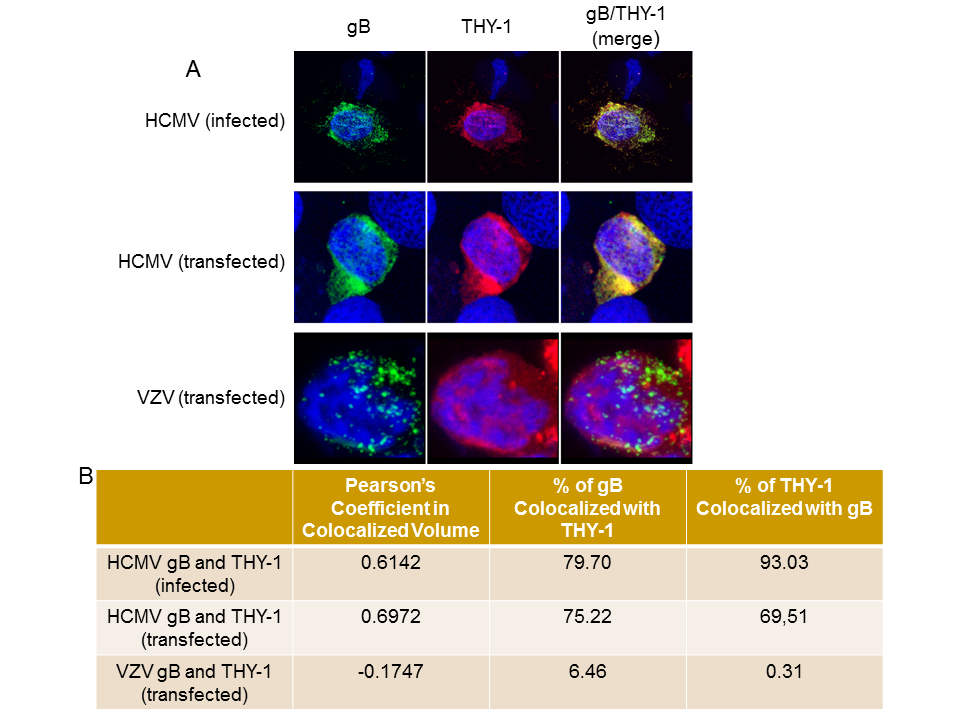

Supplement: S11 Fig — HS-578T adenocarcinoma cells were infected with HCMV AD169. 293T cells were cotransfected with the pTHY-1 expression plasmid and either a plasmid expressing HCMV gB or VZV gB. 48 hours later, the cells were stained with goat anti-THY-1, mouse anti-HCMV gB or mouse anti-VZV gB antibody followed by secondary antibodies (anti-goat Alexa 594, red, for THY-1 and anti-mouse Alexa 488, green, for gBs), and confocal microscopy and colocalization analysis were performed as described in Fig 6. (A) Z-stack images stained for HCMV and VZV gB (green) and THY-1 (red). HCMV gB colocalizes with THY-1 (yellow) in both infected and transfected cells, but VZV gB does not colocalize with THY-1. (B) Statistical analysis of colocalization. (TIF) [file ppat.1004999.s011.tif]

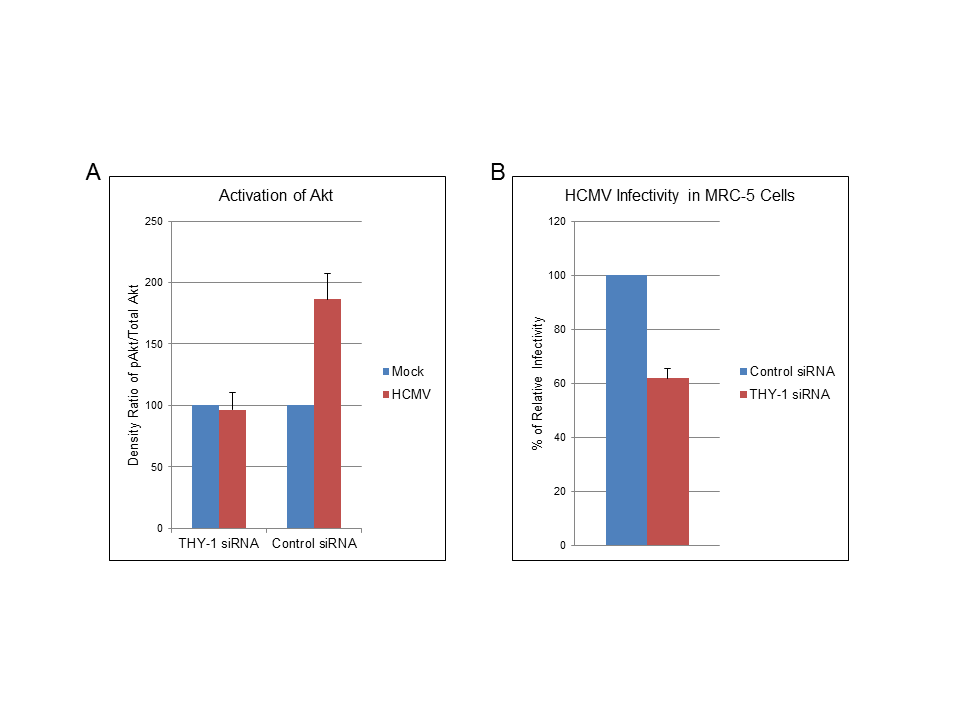

Supplement: S12 Fig — MRC-5 primary cells were nucleofected with THY-1 siRNA or control siRNA as described in Fig 8. 48 hrs after transfection, the cells were inoculated with Towne-GFP at 4°C for 60 min to allow binding and then shifted to 37°C for 15 min to allow synchronized entry. Cell lysates were prepared for immunoblots with anti-Akt and anti-phosphorylated Akt antibodies. (A) Densitometry of bands on immunoblots was quantified using ImageJ software, and the mean of the ratio of the density of pAkt/total Akt bands from six independent experiments is shown. Error bars indicate standard errors. (B) HCMV infectivity in siRNA treated MRC-5 cells. Cells were nucleofected with THY-1 siRNA or control siRNA and 48 hr later the cells were infected with CMV Towne-GFP on ice for 60 min followed by 37°C for 60 min to allow virus entry. Infectivity was scored by FACS at day 3 post-infection. The percent of relative infectivity (compared with the negative control set at 100%) from six independent experiments is shown. Error bars indicate standard errors. (TIF) [file ppat.1004999.s012.tif]
