# Supplementary material for: THY-1 Cell Surface Antigen (CD90) Has an Important Role in the Initial Stage of Human Cytomegalovirus Infection
Source: PLoS Pathog. 2015 Jul 6;11(7):e1004999. doi: 10.1371/journal.ppat.1004999 (PMC4492587; doi:10.1371/journal.ppat.1004999)
Supplement: S1 Table — Names and origins of the 54 cell lines used in the initial HCMV infectivity screen. The cell lines were purchased from Charles River Laboratories (Frederick, Maryland), and maintained in PRMI medium with 10% FBS. The cell line numbers listed are corresponding to the numbers shown in Fig 1. (DOCX) [file ppat.1004999.s013.docx]

**SUPPLEMENTAL TABLE S1. Origins of 54 human cell lines**

| **Cell line Number*** | **Cell Line Name** | **Origin** |
| --- | --- | --- |
| 1 | SF-295 | CNS Glioblastoma |
| 2 | SNB-75 | CNS Astrocytoma |
| 3 | SF-539 | CNS Gliosarcoma |
| 4 | SNB-19 | CNS Glioblastoma |
| 5 | SF-268 | CNS Astrocytoma |
| 6 | U251 | CNS Glioblastoma |
| 7 | A498 | Renal Carcinoma |
| 8 | ACHN | Renal Carcinoma |
| 9 | SN12C | Renal Carcinoma |
| 10 | UO-31 | Renal Carcinoma |
| 11 | CAKI-1 | Renal Carcinoma |
| 12 | RXF 393 | Renal Hypernephroma |
| 13 | 786-0 | Renal Adenocarcinoma |
| 14 | TK-10 | Renal Carcinoma |
| 15 | HS-578T | Ductal Carcinoma |
| 16 | MDA-MB-468 | Breast Cancer |
| 17 | BT-549 | Ductal Carcinoma |
| 18 | T-47D | Pleural Effusion Ductal Carcinoma |
| 19 | MDA-MB-231/ATCC | Pleural Effusion Adenocarcinoma |
| 20 | MCF7 | Pleural Effusion Adenocarcinoma |
| 21 | SW-620 | Colon Adenocarcinoma |
| 22 | HCT-116 | Colon Carcinoma |
| 23 | HT29 | Colon Adenocarcinoma |
| 24 | HCT-15 | Colon Adenocarcinoma |
| 25 | COLO 205 | Colon Adenocarcinoma |
| 26 | KM12 | Colon Adenocarcinoma |
| 27 | HCC-2998 | Colon Adenocarcinoma |
| 28 | DU-145 | Prostate Carcinoma |
| 29 | PC-3 | Prostate Carcinoma |
| 30 | NCI-H23 | Lung Adenocarcinoma |
| 31 | NCI-H522 | Lung Adenocarcinoma |
| 32 | EKVX | Lung Adenocarcinoma |
| 33 | NCI-H226 | Lung Squamous Carcinoma |
| 34 | A549/ATCC | Lung Adenocarcinoma |
| 35 | HOP-92 | Lung Carcinoma |
| 36 | HOP-62 | Lung Adenocarcinoma |
| 37 | NCI-H322M | Lung Carcinoma |
| 38 | NCI-H460 | Lung Carcinoma |
| 39 | M14 | Amelanotic Melanoma |
| 40 | SK-MEL-28 | Melanoma |
| 41 | UACC-62 | Melanoma |
| 42 | MDA-MB-435 | Melanoma |
| 43 | UACC-257 | Melanoma |
| 44 | SK-MEL-2 | Melanoma |
| 45 | LOX IMVI | Melanoma |
| 46 | SK-MEL-5 | Melanoma |
| 47 | MALME-3M | Melanoma |
| 48 | OVCAR-3 | Ovarian Carcinoma |
| 49 | NCI/ADR-RES | Ovarian Carcinoma |
| 50 | OVCAR-8 | Ovarian Adenocarcinoma |
| 51 | IGROV1 | Ovarian Adenocarcinoma |
| 52 | OVCAR-4 | Ovarian Adenocarcinoma |
| 53 | OVCAR-5 | Ovarian Carcinoma |
| 54 | SK-OV-3 | Ovarian Adenocarcinoma |

***** Cell line numbers, corresponding to numbers in Fig. 1
