# Supplementary material for: THY-1 Cell Surface Antigen (CD90) Has an Important Role in the Initial Stage of Human Cytomegalovirus Infection
Source: PLoS Pathog. 2015 Jul 6;11(7):e1004999. doi: 10.1371/journal.ppat.1004999 (PMC4492587; doi:10.1371/journal.ppat.1004999)
Supplement: S2 Table — Representative raw FACS data of HCMV susceptibility in 54 cell lines. Only one set of the multiple infections of 10 cell lines is shown. (DOCX) [file ppat.1004999.s014.docx]

Raw data from NCI-60 screen (first 10 cell lines)

| **Cell lines** | **Depth** |  | **Name** | **Statistic** | **#Cells** | **Infected - Mock (Q3)** | **% of ARPE-19** |
| --- | --- | --- | --- | --- | --- | --- | --- |
| ARPE-19 |  |  | 1.001 |  | 24450 |  |  |
|  | > |  | Q1: FL1-Height- , FSC-Height+ | 0 | 0 |  |  |
|  | > |  | Q2: FL1-Height+ , FSC-Height+ | 0 | 0 |  |  |
|  | > | Mock | Q3: FL1-Height+ , FSC-Height- | 0.147 | 36 |  |  |
|  | > |  | Q4: FL1-Height- , FSC-Height- | 99.9 | 24414 |  |  |
|  |  |  | 1.002 |  | 34095 |  |  |
|  | > |  | Q1: FL1-Height- , FSC-Height+ | 0 | 0 |  |  |
|  | > |  | Q2: FL1-Height+ , FSC-Height+ | 0 | 0 |  |  |
|  | > | HCMV | Q3: FL1-Height+ , FSC-Height- | 2.04 | 697 | 1.893 | 100 |
|  | > |  | Q4: FL1-Height- , FSC-Height- | 98 | 33398 |  |  |
| SNB-75 |  |  | 1.003 |  | 33210 |  |  |
|  | > |  | Q1: FL1-Height- , FSC-Height+ | 0 | 0 |  |  |
|  | > |  | Q2: FL1-Height+ , FSC-Height+ | 0 | 0 |  |  |
|  | > | Mock | Q3: FL1-Height+ , FSC-Height- | 0.081 | 27 |  |  |
|  | > |  | Q4: FL1-Height- , FSC-Height- | 99.9 | 33183 |  |  |
|  |  |  | 1.004 |  | 28305 |  |  |
|  | > |  | Q1: FL1-Height- , FSC-Height+ | 0 | 0 |  |  |
|  | > |  | Q2: FL1-Height+ , FSC-Height+ | 0 | 0 |  |  |
|  | > | HCMV | Q3: FL1-Height+ , FSC-Height- | 0.692 | 196 | 0.611 | 32.2768093 |
|  | > |  | Q4: FL1-Height- , FSC-Height- | 99.3 | 28109 |  |  |
| NCI-H522 |  |  | 1.005 |  | 42870 |  |  |
|  | > |  | Q1: FL1-Height- , FSC-Height+ | 0 | 0 |  |  |
|  | > |  | Q2: FL1-Height+ , FSC-Height+ | 0 | 0 |  |  |
|  | > |  | Q3: FL1-Height+ , FSC-Height- | 0.082 | 35 |  |  |
|  | > |  | Q4: FL1-Height- , FSC-Height- | 99.9 | 42835 |  |  |
|  |  |  | 1.006 |  | 55920 |  |  |
|  | > |  | Q1: FL1-Height- , FSC-Height+ | 0 | 0 |  |  |
|  | > |  | Q2: FL1-Height+ , FSC-Height+ | 0 | 0 |  |  |
|  | > | Mock | Q3: FL1-Height+ , FSC-Height- | 0.633 | 354 | 0.551 | 29.10723719 |
|  | > |  | Q4: FL1-Height- , FSC-Height- | 99.4 | 55566 |  |  |
| T-47D |  |  | 1.007 |  | 38805 |  |  |
|  | > |  | Q1: FL1-Height- , FSC-Height+ | 0 | 0 |  |  |
|  | > |  | Q2: FL1-Height+ , FSC-Height+ | 0 | 0 |  |  |
|  | > | HCMV | Q3: FL1-Height+ , FSC-Height- | 0.247 | 96 |  |  |
|  | > |  | Q4: FL1-Height- , FSC-Height- | 99.8 | 38709 |  |  |
|  |  |  | 1.008 |  | 51390 |  |  |
|  | > |  | Q1: FL1-Height- , FSC-Height+ | 0 | 0 |  |  |
|  | > |  | Q2: FL1-Height+ , FSC-Height+ | 0 | 0 |  |  |
|  | > |  | Q3: FL1-Height+ , FSC-Height- | 0.872 | 448 | 0.625 | 33.01637612 |
|  | > |  | Q4: FL1-Height- , FSC-Height- | 99.1 | 50942 |  |  |
| NCI/ADR-RES |  |  | 1.009 |  | 256740 |  |  |
|  | > |  | Q1: FL1-Height- , FSC-Height+ | 0 | 0 |  |  |
|  | > |  | Q2: FL1-Height+ , FSC-Height+ | 0 | 0 |  |  |
|  | > | Mock | Q3: FL1-Height+ , FSC-Height- | 0.004 | 11 |  |  |
|  | > |  | Q4: FL1-Height- , FSC-Height- | 100 | 256729 |  |  |
|  |  |  | 1.01 |  | 207210 |  |  |
|  | > |  | Q1: FL1-Height- , FSC-Height+ | 0 | 0 |  |  |
|  | > |  | Q2: FL1-Height+ , FSC-Height+ | 0 | 0 |  |  |
|  | > | HCMV | Q3: FL1-Height+ , FSC-Height- | 0.2 | 415 | 0.196 | 10.35393555 |
|  | > |  | Q4: FL1-Height- , FSC-Height- | 99.8 | 206795 |  |  |
| SF-295 |  |  | 1.011 |  | 231375 |  |  |
|  | > |  | Q1: FL1-Height- , Side Scatter+ | 0 | 0 |  |  |
|  | > |  | Q2: FL1-Height+ , Side Scatter+ | 0 | 0 |  |  |
|  | > | Mock | Q3: FL1-Height+ , Side Scatter- | 0.015 | 34 |  |  |
|  | > |  | Q4: FL1-Height- , Side Scatter- | 100 | 231341 |  |  |
|  |  |  | 1.012 |  | 165480 |  |  |
|  | > |  | Q1: FL1-Height- , Side Scatter+ | 0 | 0 |  |  |
|  | > |  | Q2: FL1-Height+ , Side Scatter+ | 0 | 0 |  |  |
|  | > | HCMV | Q3: FL1-Height+ , Side Scatter- | 0.036 | 60 | 0.021 | 1.109350238 |
|  | > |  | Q4: FL1-Height- , Side Scatter- | 100 | 165420 |  |  |
| SN12C |  |  | 1.013 |  | 230655 |  |  |
|  | > |  | Q1: FL1-Height- , Side Scatter+ | 0 | 0 |  |  |
|  | > |  | Q2: FL1-Height+ , Side Scatter+ | 0 | 0 |  |  |
|  | > | Mock | Q3: FL1-Height+ , Side Scatter- | 0.187 | 431 |  |  |
|  | > |  | Q4: FL1-Height- , Side Scatter- | 99.8 | 230224 |  |  |
|  |  |  | 1.014 |  | 249990 |  |  |
|  | > |  | Q1: FL1-Height- , Side Scatter+ | 0 | 0 |  |  |
|  | > |  | Q2: FL1-Height+ , Side Scatter+ | 0 | 0 |  |  |
|  | > | HCMV | Q3: FL1-Height+ , Side Scatter- | 0.239 | 598 | 0.052 | 2.746962493 |
|  | > |  | Q4: FL1-Height- , Side Scatter- | 99.8 | 249392 |  |  |
| A549/ATCC |  |  | 1.015 |  | 128025 |  |  |
|  | > |  | Q1: FL1-Height- , Side Scatter+ | 0 | 0 |  |  |
|  | > |  | Q2: FL1-Height+ , Side Scatter+ | 0 | 0 |  |  |
|  | > | Mock | Q3: FL1-Height+ , Side Scatter- | 1.78 | 2283 |  |  |
|  | > |  | Q4: FL1-Height- , Side Scatter- | 98.2 | 125742 |  |  |
|  |  |  | 1.016 |  | 256515 |  |  |
|  | > |  | Q1: FL1-Height- , Side Scatter+ | 0 | 0 |  |  |
|  | > |  | Q2: FL1-Height+ , Side Scatter+ | 0 | 0 |  |  |
|  | > | HCMV | Q3: FL1-Height+ , Side Scatter- | 2.26 | 5789 | 0.48 | 25.35657686 |
|  | > |  | Q4: FL1-Height- , Side Scatter- | 97.7 | 250726 |  |  |
| OVCAR-8 |  |  | 1.017 |  | 341205 |  |  |
|  | > |  | Q1: FL1-Height- , Side Scatter+ | 0 | 0 |  |  |
|  | > |  | Q2: FL1-Height+ , Side Scatter+ | 0 | 0 |  |  |
|  | > | Mock | Q3: FL1-Height+ , Side Scatter- | 0.047 | 162 |  |  |
|  | > |  | Q4: FL1-Height- , Side Scatter- | 100 | 341043 |  |  |
|  |  |  | 1.018 |  | 319110 |  |  |
|  | > |  | Q1: FL1-Height- , Side Scatter+ | 0 | 0 |  |  |
|  | > |  | Q2: FL1-Height+ , Side Scatter+ | 0 | 0 |  |  |
|  | > | HCMV | Q3: FL1-Height+ , Side Scatter- | 0.054 | 171 | 0.007 | 0.369783413 |
|  | > |  | Q4: FL1-Height- , Side Scatter- | 99.9 | 318939 |  |  |
| LOX IMVI |  |  | 1.019 |  | 145545 |  |  |
|  | > |  | Q1: FL1-Height- , Side Scatter+ | 0 | 0 |  |  |
|  | > |  | Q2: FL1-Height+ , Side Scatter+ | 0 | 0 |  |  |
|  | > | Mock | Q3: FL1-Height+ , Side Scatter- | 0.066 | 96 |  |  |
|  | > |  | Q4: FL1-Height- , Side Scatter- | 99.9 | 145449 |  |  |
|  |  |  | 1.02 |  | 128760 |  |  |
|  | > |  | Q1: FL1-Height- , Side Scatter+ | 0 | 0 |  |  |
|  | > |  | Q2: FL1-Height+ , Side Scatter+ | 0 | 0 |  |  |
|  | > | HCMV | Q3: FL1-Height+ , Side Scatter- | 0.12 | 155 | 0.054 | 2.852614897 |
|  | > |  | Q4: FL1-Height- , Side Scatter- | 99.9 | 128605 |  |  |
| SW-620 |  |  | 1.021 |  | 421350 |  |  |
|  | > |  | Q1: FL1-Height- , Side Scatter+ | 0 | 0 |  |  |
|  | > |  | Q2: FL1-Height+ , Side Scatter+ | 0 | 0 |  |  |
|  | > | Mock | Q3: FL1-Height+ , Side Scatter- | 0.011 | 45 |  |  |
|  | > |  | Q4: FL1-Height- , Side Scatter- | 100 | 421305 |  |  |
|  |  |  | 1.022 |  | 567060 |  |  |
|  | > |  | Q1: FL1-Height- , Side Scatter+ | 0 | 0 |  |  |
|  | > |  | Q2: FL1-Height+ , Side Scatter+ | 0 | 0 |  |  |
|  | > | HCMV | Q3: FL1-Height+ , Side Scatter- | 0.005 | 29 | -0.006 | -0.003169572 |
|  | > |  | Q4: FL1-Height- , Side Scatter- | 100 | 567031 |  |  |
