# Supplementary material for: THY-1 Cell Surface Antigen (CD90) Has an Important Role in the Initial Stage of Human Cytomegalovirus Infection
Source: PLoS Pathog. 2015 Jul 6;11(7):e1004999. doi: 10.1371/journal.ppat.1004999 (PMC4492587; doi:10.1371/journal.ppat.1004999)
Supplement: S3 Table — HCMV encoded proteins identified by mass spectrometry from the pull-down assay by anti-THY-1 antibody. Only those with ΣCoverage score greater than 2.0 were shown (ΣCoverage score measures the number of peptides recovered for each protein and the protein coverage. The threshold for statistical significance in the bioinformatics analysis was set at 0.5). (DOCX) [file ppat.1004999.s015.docx]

| Accession | # AAs | MW [kDa] | Description | ΣCoverage | Σ# PSMs | Σ# Peptides | Score A(2,3) | Coverage A(2,3) |
| --- | --- | --- | --- | --- | --- | --- | --- | --- |
| B9VXP1_HCMV | 561 | 62.84244 | (B9VXP1) Tegument protein pp65 | 50.09 | 110 | 19 | 6561.99884 | 50.09 |
| B9VXP4_HCMV | 1370 | 153.6862 | (B9VXP4) Major capsid protein | 31.9 | 71 | 30 | 4099.63545 | 31.9 |
| B9VXJ6_HCMV | 656 | 73.46584 | (B9VXJ6) Tegument protein UL25 | 28.05 | 31 | 11 | 1599.80259 | 28.05 |
| A8T7G0_HCMV | 559 | 61.96371 | (A8T7G0) Tegument protein pp71 | 16.64 | 9 | 7 | 510.094914 | 16.64 |
| Q9DXH5_HCMV | 1046 | 112.5862 | (Q9DXH5) UL32 | 11.38 | 17 | 10 | 1359.74945 | 11.38 |
| D2W9M3_HCMV | 164 | 17.03643 | (D2W9M3) Membrane glycoprotein UL119 | 10.37 | 1 | 1 | 57.94 | 10.37 |
| Q69170_HCMV | 906 | 101.7897 | (Q69170) Envelope glycoprotein B | 9.93 | 31 | 6 | 1140.56798 | 9.93 |
| B9VXQ6_HCMV | 190 | 20.9373 | (B9VXQ6) Myristylated tegument protein | 8.95 | 1 | 1 | 61.5320456 | 8.95 |
| B9VXN9_HCMV | 372 | 38.11184 | (B9VXN9) Capsid scaffold protein | 6.99 | 11 | 2 | 394.398668 | 6.99 |
| A1IKL9_HCMV | 742 | 84.25819 | (A1IKL9) Glycoprotein H | 6.74 | 7 | 4 | 280.998607 | 6.74 |
| D2K3X7_HCMV | 219 | 25.64629 | (D2K3X7) Chloramphenicol acetyltransferase | 6.39 | 1 | 1 | 111.01 | 6.39 |
| B9VXL6_HCMV | 2240 | 253.0792 | (B9VXL6) Large tegument protein | 5.94 | 15 | 11 | 935.42132 | 5.94 |
| B8YE71_HCMV | 640 | 72.48395 | (B8YE71) Tegument protein UL35 | 5 | 2 | 2 | 159.236648 | 5 |
| B9VXQ7_HCMV | 372 | 42.8045 | (B9VXQ7) Envelope glycoprotein M | 4.3 | 7 | 1 | 382.487741 | 4.3 |
| D2K4T7_HCMV | 793 | 84.38207 | (D2K4T7) Tegument protein TRS1 | 4.29 | 3 | 3 | 310.137045 | 4.29 |
| C8CPG4_HCMV | 457 | 53.22622 | (C8CPG4) Envelope glycoprotein O | 4.16 | 2 | 1 | 127.975146 | 4.16 |
| C8CPF1_HCMV | 396 | 42.76666 | (C8CPF1) Nuclear egress membrane protein | 3.79 | 7 | 1 | 405.405556 | 3.79 |
| D3YS09_HCMV | 306 | 34.57228 | (D3YS09) Capsid triplex subunit 2 | 3.59 | 1 | 1 | 82.4758885 | 3.59 |
| B9VXL5_HCMV | 983 | 109.982 | (B9VXL5) Tegument protein UL37 | 2.03 | 2 | 2 | 185.231395 | 2.03 |
